# Manipulation of the through‐space interactions in diphenylmethane

**DOI:** 10.1002/smo.20220006

**Published:** 2023-03-20

**Authors:** Weihao Tu, Zuping Xiong, Ziteng Zhang, Jianyu Zhang, Lei Wang, Yuan Xie, Yipu Wang, Haoke Zhang, Jing Zhi Sun, Ben Zhong Tang

**Affiliations:** ^1^ MOE Key Laboratory of Macromolecular Synthesis and Functionalization Department of Polymer Science and Engineering Zhejiang University Hangzhou China; ^2^ ZJU‐Hangzhou Global Scientific and Technological Innovation Center Hangzhou China; ^3^ Department of Chemistry Hong Kong Branch of Chinese National Engineering Research Center for Tissue Restoration and Reconstruction The Hong Kong University of Science and Technology Hong Kong China; ^4^ Center for AIE Research College of Materials Science and Engineering Shenzhen University Shenzhen China; ^5^ School of Science and Engineering Shenzhen Institute of Aggregate Science and Technology The Chinese University of Hong Kong Shenzhen China

**Keywords:** clusteroluminescence, diphenylmethane, photophysics, through‐space interactions

## Abstract

Through‐space interaction (TSI) has been proven to play an important role in the newly emerging clusteroluminescence (CL) phenomenon. However, it is still a big challenge to manipulate the TSI at the molecular level due to the unclear relationship between the non‐conjugated structure and TSI properties. Herein, the TSI in diphenylmethane is manipulated by breaking its symmetric structures and changing the isolated subunits. Finally, the CL wavelength and efficiency of diphenylmethane are successfully regulated at the aggregate state.

## Graphical abstract

This work not only demonstrates the through‐space interaction (TSI) in diphenylmethane (DPM) but also provides new strategies for the manipulation of TSI and clusteroluminescence in DPM derivatives by breaking the symmetric structures and changing the isolated subunits.

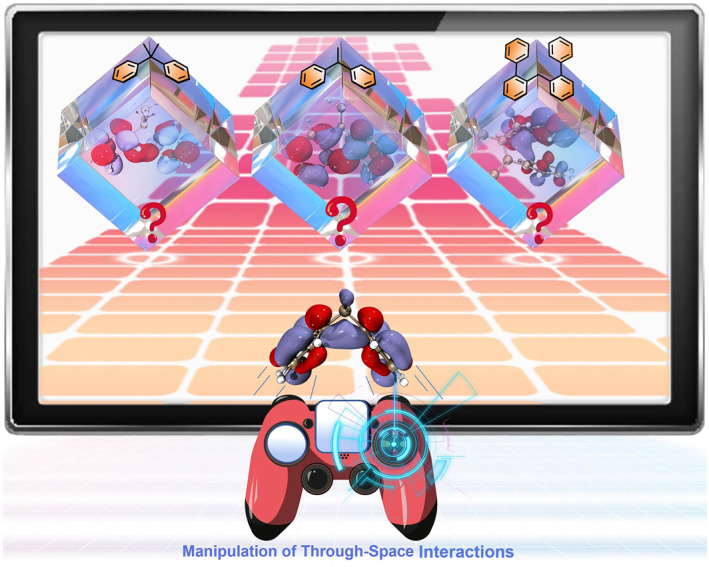

## 1 INTRODUCTION

Since the concept of fluorescence was coined by George Gabriel Stokes in 1852, people have summarized various strategies to improve the photophysical performance of luminescent materials with π‐conjugated structures, such as regulating the π‐conjugated length and introducing the intramolecular charge transfer state, the excited state molecular planarization, and the excited‐state intramolecular proton transfer.[^1^, ^2^, ^3^, ^4^] Within the past one and a half centuries, the corresponding theory of molecular photophysics based on through‐bond conjugation (TBC) has been established to guide the design of functional luminescent materials.[^5^, ^6^, ^7^, ^8^, ^9^] However, recently, more and more non‐conjugated molecules, such as tetraphenylethane, maleimide, and polystyrene, were discovered with abnormal bright visible emissions at the clustering state, which is named clusteroluminescence (CL), and the corresponding process is described as clusterization‐triggered emission (CTE).[^10^, ^11^, ^12^, ^13^, ^14^] However, the traditional TBC‐based photophysical theory is unable to explain the CL. So, it is urgent to establish a rational theory to explain such abnormal photoluminescence (PL).

Recently, different from the TBC effect in π‐conjugated chromophores, through‐space interaction (TSI) was proven to play a crucial role in the CL generation, including through‐space *n*···*n*, *n*···π and π···π interactions.[^15^, ^16^, ^17^] Generally, TSI could be considered to be a kind of electronic interaction, which may come from both intermolecular and intramolecular interactions.[^18^, ^19^] Apart from the mechanistic exploration of CL, developing efficient CL luminogens (CLgens) with adjustable emission wavelength is also a big challenge in this area due to the unclear structure–property relationship. Compared with the polymer‐based CLgens with ambiguous conformation, non‐conjugated small molecules with precise structures are the best candidates to solve the above‐mentioned two problems: unclear TSI mechanism and the manipulation of CL properties. According to the previous reports, among all the non‐conjugated small molecules with CL, diphenylmethane (DPM) is the simplest to exhibit typical TSI (homoconjugation), which could be easily modified at different positions.[^20^, ^21^] Therefore, it is expected that a systematic structural modification and photophysical characterization of DPM could reveal its intrinsic TSI mechanism and help to build a relationship between conformation and CL properties.

Based on these considerations, herein, four DPM derivatives were synthesized, purified, and fully characterized via nuclear magnetic resonance (NMR), gas‐chromatography mass spectrometry (GC‐MS), and high‐performance liquid chromatography (HPLC) (Figures S1–S12). Experimental characterization and theoretical calculation results proved that DPM showed TSI and CL, which could be regulated by adding one methyl group in the center SP^3^ carbon. By changing the molecular symmetry, its CL efficiency was improved and the emission peak became narrower. Meanwhile, decorating another benzene ring in the ortho‐position of DPM could increase the TSI and lead to a redshifted CL. This work provides two effective methods to regulate the photophysical performance of DPM via changing the intramolecular TSI, which would further enrich the TSI theory for CL.

## 2 RESULTS AND DISCUSSION

**FIGURE 1:**
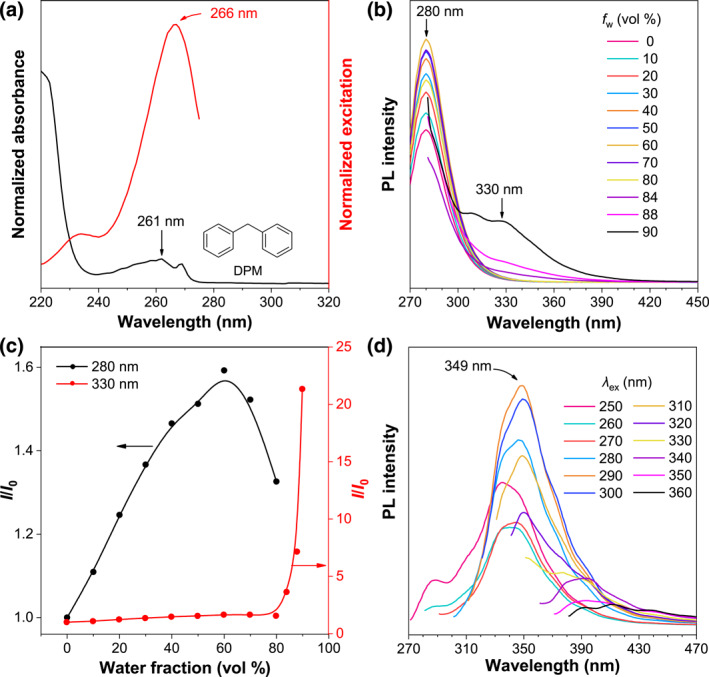
(a) Absorption and excitation spectra of DPM in ACN solution. Concentration (*c*): 10^−4^ M, *λ*_em_ = 280 nm for the excitation spectrum. (b) PL spectra of DPM in ACN/water mixtures with different water fractions (*f*_*w*_). *c* = 5 × 10^−4^ M and *λ*_ex_ = 260 nm. (c) Plots of relative PL intensity (*I*/*I*_0_) versus *f*_*w*_ and *I*_0_ = intensity at *f*_*w*_ = 0%. (d) PL spectra with different excitation wavelengths (*λ*_ex_) of pure DPM liquid. ACN, acetonitrile; DPM, diphenylmethane; PL, Photoluminescence.

DPM is a typical non‐conjugated small molecule with two isolated benzenes, which was proved by its absorption spectrum in acetonitrile (ACN). As shown in Figure 1a, the absorption maximum (*λ*_ab_) of DPM located at 261 nm corresponded to the isolated benzene ring, indicating the absence of TBC in DPM. Meanwhile, the excitation spectrum has a similar maximum peak at 266 nm under the emission wavelength of 280 nm which is the maximum emission (*λ*_em_) of benzene.^[22]^ Then the PL spectra of DPM in ACN/water mixture were recorded at different water fractions (*f*_*w*_). As shown in Figure 1b, at low *f*_*w*_ (0%–80%), an obvious emission peak corresponding to the isolated benzene ring at 280 nm was observed. It is noteworthy that during the aggregation process, the emission peak at 280 nm first increased and then decreased, and the turning point was located at *f*_*w*_ = 60%. According to our previous report, the PL intensity variation at 280 nm was caused by the changing of solvent polarity and aggregation effect.^[21]^ With further increasing *f*_*w*_, a new broad emission peak in the range of 300–390 nm appeared, and its intensity at *f*_*w*_ = 90% improved 22 times more than that at *f*_*w*_ = 0% (Figure 1c). The emerging long‐wavelength emission is CL, which is induced by the stable intramolecular TSI once forming the aggregates.[^23^, ^24^] During the whole process, with the addition of water, the increased solvent polarity improved the TBC emission. However, when the *f*_*w*_ was above 60%, the aggregation gradually occurred and the internal polarity of aggregates was less affected by the surrounding environment. Moreover, the short‐wavelength TBC emission was transferred into the TSI state via Förster resonance energy transfer (FRET), thus leading to the decrease of TBC emission and the increase of TSI emission. In Figure 1d, PL spectra of pure DPM liquid exhibited two emission peaks at 287 and 349 nm, respectively, under the excitation of 250 nm, which also corresponded to the TBC and TSI emission of DPM. Then, the TBC emission was weakened and disappeared with increasing the excitation wavelength (*λ*_ex_), and the TSI emission at 349 nm reached the maximum at *λ*_ex_ = 310 nm. When the *λ*_ex_ increased from 310 to 360 nm, the TSI emission showed redshifted and the intensity was decreased. For example, at *λ*_ex_ = 360 nm, the *λ*_em_ located around 410 nm with quite low intensity. The above results suggested that different DPM conformations with variable TSI existed in the solid state but the conformation corresponding to the 349 nm emission should have the most probability of distribution.

**FIGURE 2:**
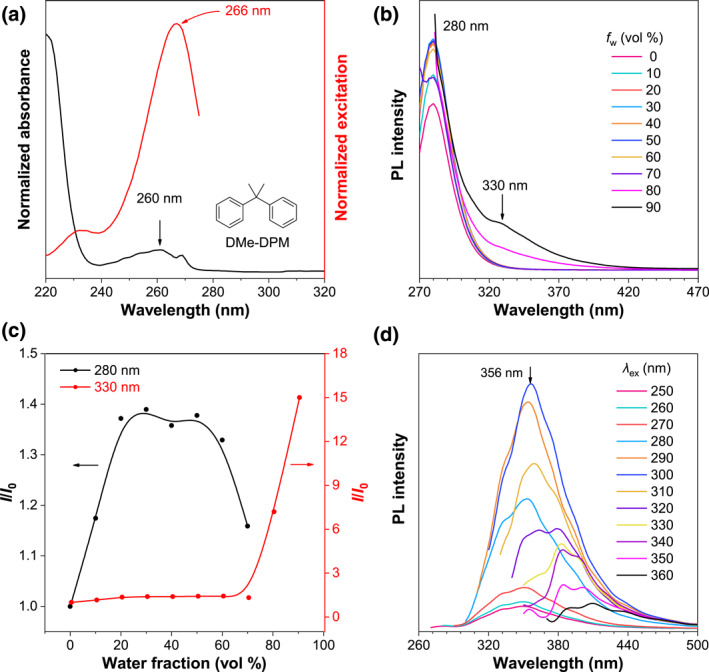
(a) Absorption and excitation spectra of DMe‐DPM in ACN solution. Concentration (*c*): 10^−4^ M, *λ*_em_ = 280 nm for the excitation spectrum. (b) PL spectra of DMe‐DPM in ACN/water mixtures with different water fractions (*f*_*w*_). *c* = 5 × 10^−4^ M, *λ*_ex_ = 260 nm. (c) Plots of relative PL intensity (*I*/*I*_0_) versus *f*_*w*_, *I*_0_ = intensity at *f*_*w*_ = 0%. (d) PL spectra with different excitation wavelengths (*λ*_ex_) of pure DMe‐DPM liquid. ACN, acetonitrile; DMe‐DPM, propane‐2,2‐diyldibenzene; PL, Photoluminescence.

Since the CL in DPM comes from the homoconjugation between two benzene rings, it is proposed that such TSI could be manipulated by changing the conformation of DPM. Therefore, two methyl groups were symmetrically added to its central carbon atom to generate propane‐2,2‐diyldibenzene (DMe‐DPM). Photophysical characterization on DMe‐DPM indicated that it showed similar absorption and excitation spectra with DPM (Figure 2a). The PL spectra in ACN/water mixture were recorded for DMe‐DPM, which also showed a broad TSI emission in the range of 300–390 nm. However, different from DPM, the emission intensity at 280 nm reached the maximum at *f*_*w*_ = 20%, and TSI emission was induced once the *f*_*w*_ was above 70% which is earlier than DPM, indicating that the TSI is easier to be stabilized in DMe‐DPM because of its poorer solubility in ACN than that of DPM (Figure 2b,c). Moreover, DMe‐DPM showed a slightly redshifted CL with *λ*_em_ = 356 nm, and the TBC emission was almost undetectable in its bulk liquid (Figure 2d). The photophysical performance of DMe‐DPE suggested that the introduction of two methyl groups did not change the TSI between two isolated benzene rings too much.

**FIGURE 3:**
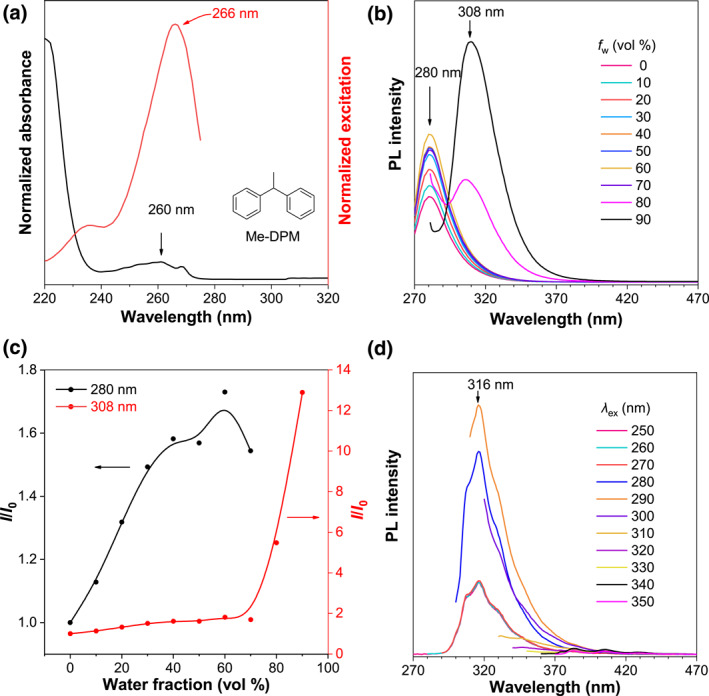
(a) Absorption and excitation spectra of Me‐DPM in ACN solution. Concentration (*c*): 10^−4^ M, *λ*_em_ = 280 nm for the excitation spectrum. (b) PL spectra of Me‐DPM in ACN/water mixtures with different water fractions (*f*_*w*_). *c* = 5 × 10^−4^ M, *λ*_ex_ = 260 nm. (c) Plots of relative PL intensity (*I*/*I*_0_) versus *f*_*w*_, *I*_0_ = intensity at *f*_*w*_ = 0%. (d) PL spectra with different excitation wavelengths (*λ*_ex_) of pure Me‐DPM liquid. ACN, acetonitrile; Me‐DPM, ethane‐1,1‐diyldibenzene; PL, Photoluminescence.

As mentioned above, adding two symmetric methyl groups in central carbon could not effectively manipulate the TSI in DPM; then, we were wondering how the molecular symmetry will affect the TSI. The photophysical properties of ethane‐1,1‐diyldibenzene (Me‐DPM) were systematically studied and the results were shown in Figure 3. Similar to DPM and DMe‐DPM, Me‐DPM showed the same absorption and excitation spectra in its ACN solution (Figure 3a). However, Me‐DPM exhibited different PL properties with DPM and DMe‐DPM. As shown in Figure 2b, when the *f*_*w*_ is above 70%, a sharp and intensified TSI emission was observed at 308 nm, which is blueshifted than the other two structures (Figure 3b,c). For the bulk liquid, the TSI emission is located at 316 nm, which is also shorter but sharper than DPM and DMe‐DPM (Figure 3d). Meanwhile, the fluorescence quantum yield (QY) of DPM and DMe‐DPM was undetectable (<1%) but Me‐DPM showed about 6% QY at *f*_*w*_ = 90%. The above results indicated that the asymmetric structure could stabilize the TSI and increase QY, but the weakened TSI decreased the *λ*_em_.

**FIGURE 4:**
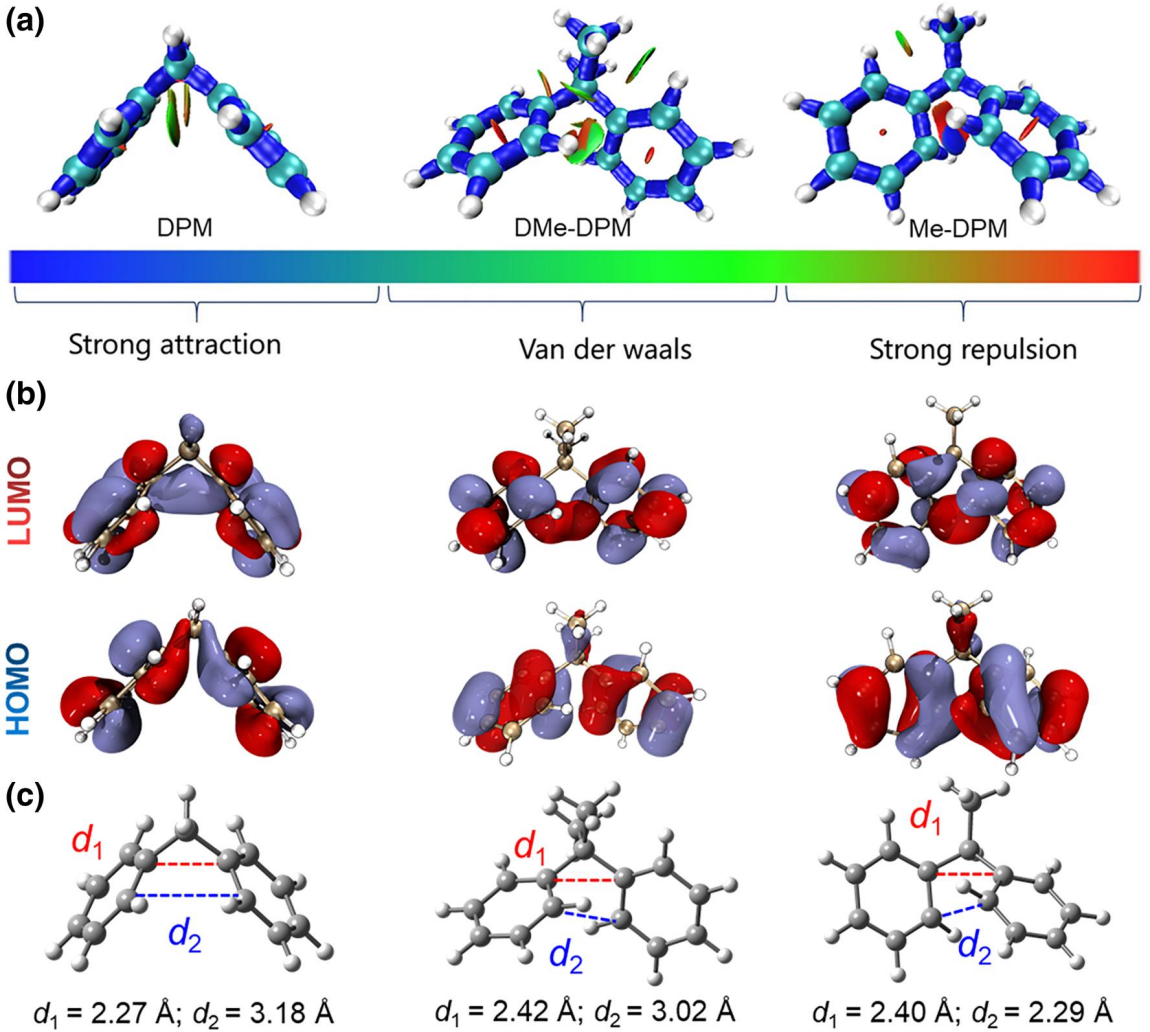
(a) Isosurface map of interaction region indicator of optimized excited‐state geometries of DPM, DMe‐DPM, and Me‐DPM (gas phase). (b) Frontier molecular orbitals of optimized excited‐state geometries of DPM, DMe‐DPM, and Me‐DPM (gas phase). (c) The optimized excited‐state geometries of DPM, DMe‐DPM, and Me‐DPM (gas phase). The results were calculated by the TD‐DFT method at the B3LYP‐D3/6‐31G(d,p) level, Gaussian 09 program. DPM, diphenylmethane; TD‐DFT, time‐dependent density functional theory.

To reveal the relationship between DPM conformation and the TSI, the single‐molecule geometry of three molecules in the ground and excited states were optimized, and their electronic structures were calculated by the density functional theory (DFT) method at the B3LYP‐D3/6‐31G(d,p) level (Table S1–S8). Figure 4a showed the isosurface map of the interaction region indicator (IRI) for these DPM derivatives at the excited state. Homoconjugation was always observed between two isolated benzene rings, but DPM and DMe‐DPM performed the Van der Waals interaction, and Me‐DPM exhibited a strong attraction. Meanwhile, the frontier molecular orbitals analysis directly demonstrated the TSI in the lowest unoccupied molecular orbital (LUMO), which is the origin of the CL in these DPM derivatives (Figure 4b). Figure 4c showed the optimized conformation of these molecules and the intramolecular distances (*d*_1_,*d*_2_) between two close carbons in the separated benzene rings were measured. As expected, the *d*_1_ and *d*_2_ in DPM and DMe‐DPM were quite close (2.27 Å, 3.18 Å) and (2.42 Å, 3.02 Å), respectively. However, *d*_1_ and *d*_2_ of Me‐DPM were 2.40 Å and 2.29 Å, respectively. Obviously, the *d*_2_ decreased a lot in this asymmetric structure, which is the origin of the strong attraction and stabilized TSI in Me‐DPM. Because of the stabilized conformation, the area of electron overlap between two benzene rings was decreased, resulting in the blueshifted TSI emission.

**FIGURE 5:**
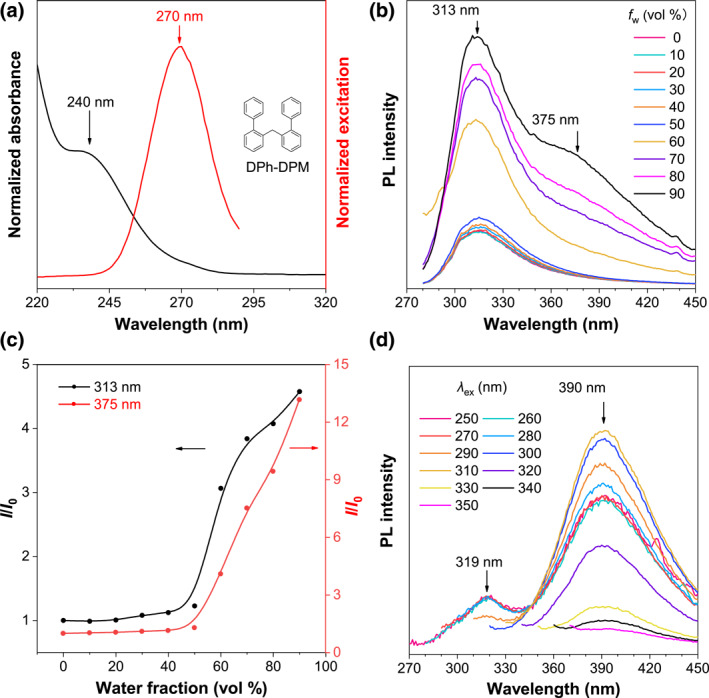
(a) Absorption and excitation spectra of DPh‐DPM in ACN solution. Concentration (*c*): 10^−4^ M, *λ*_em_ = 313 nm for the excitation spectrum. (b) PL spectra of DPh‐DPM in ACN/water mixtures with different water fractions (*f*_*w*_). *c* = 5 × 10^−4^ M and *λ*_ex_ = 270 nm. (c) Plots of relative PL intensity (*I*/*I*_0_) versus *f*_*w*_, *I*_0_ = intensity at *f*_*w*_ = 0%. (d) Solid‐state PL spectra of DPh‐DPM with different excitation wavelengths (*λ*_ex_). ACN, acetonitrile; DPh‐DPM, di([1,1′‐biphenyl]‐2‐yl)methane; PL, Photoluminescence.

Introducing one bulky group in the central SP^3^ carbon‐generated asymmetric Me‐DPM, which could stabilize but weaken the intramolecular TSI. Then, we were wondering how the TSI will be regulated if the bulky groups were decorated in the bilateral benzene rings instead of the central carbon. Considering the negligible effect of a methyl group on the outer phenyl rings, two other phenyl rings were selected to modify the DPM at the ortho‐position and di([1,1′‐biphenyl]‐2‐yl)methane (DPh‐DPM) was synthesized. As shown in Figure 5a, DPh‐DPM showed a broad and strong absorption peak with *λ*_ab_ = 240 nm but the tail extended to 290 nm; meanwhile, its excitation spectrum exhibited a slightly redshifted peak at 270 nm, which corresponded to the electronic transition of biphenyl subunit, indicating the non‐conjugated structure of DPh‐DPM. Afterward, its PL spectra at ACN/water mixtures were recorded under different *f*_*w*_. Figure 5b suggested that DPh‐DPM possessed a 313 nm emission at *f*_*w*_ ≦ 50%, which originated from the intrinsic fluorescence of biphenyl. Once the *f*_*w*_ was above 50%, the aggregates were gradually formed and a broad peak appeared at the long‐wavelength range with *λ*_em_ = 375 nm. Different from the above three DPM derivates, both the short‐wavelength TBC and long‐wavelength TSI emission were intensified when forming the aggregates at *f*_*w*_ > 50%, which should be ascribed to the synergistic effect between energy transfer and restriction of intramolecular motion (RIM) for the biphenyl (Figure 5c).^[25]^ The solid‐state PL spectra for DPh‐DPM were measured, which also showed two emission peaks under different *λ*_ex_, the TBC and TSI peaks at 319 and 390 nm, respectively (Figure 5d). In comparison with the above three DPM derivatives, DPh‐DPM exhibited the largest wavelength gap between TBC and TSI emission, 71 nm, suggesting the more efficient intramolecular TSI in DPh‐DPM.

**FIGURE 6:**
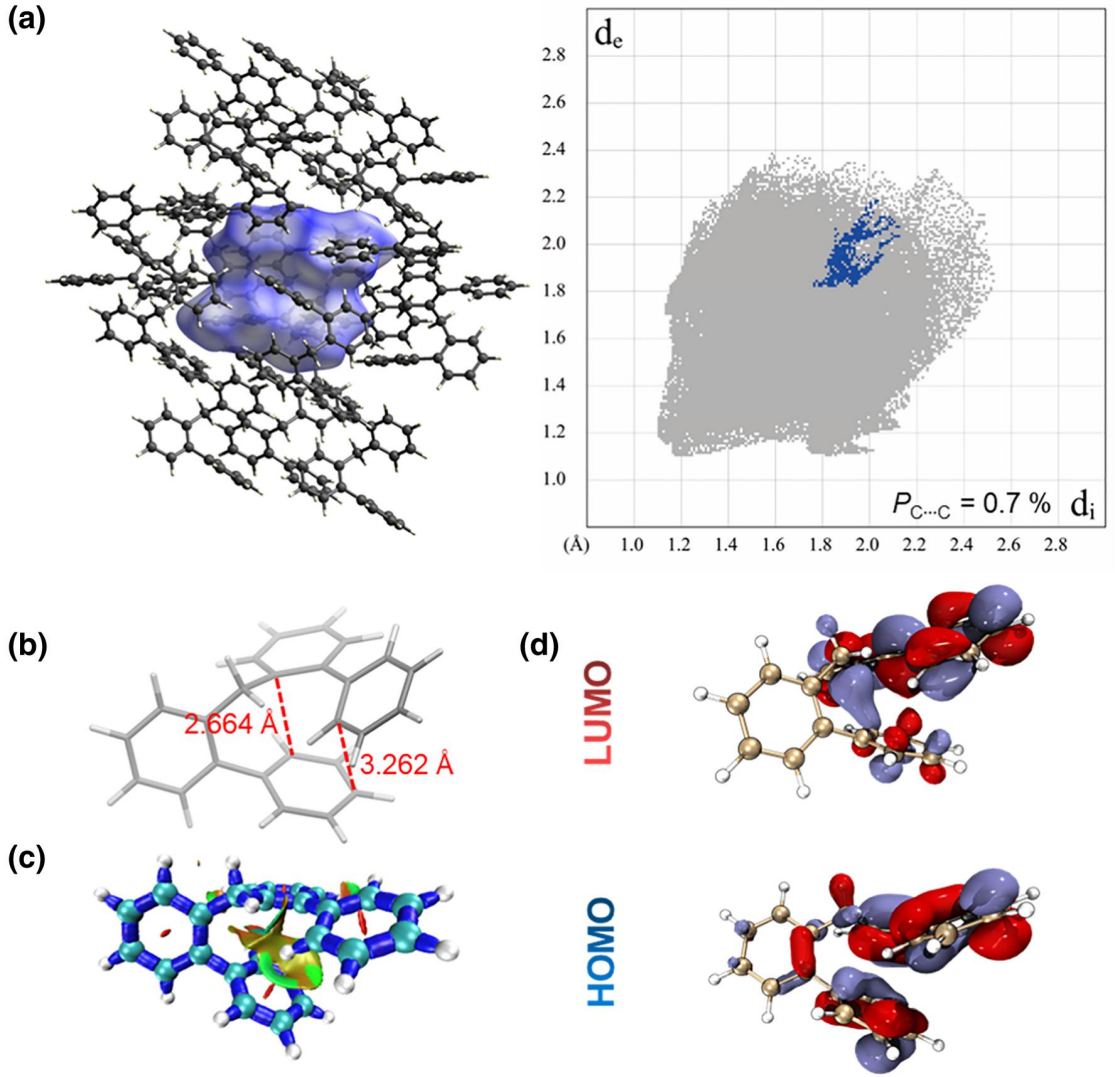
(a) Hirshfeld surfaces (mapped over *d*_norm_) and decomposed fingerprint plots of DPh‐DPM. The full fingerprints appear as gray shadows underneath decomposed plots, and the intermolecular C···C interaction is shown as a blue shadow. *P*_C···C_: proportion of intermolecular C···C interaction to total intermolecular interaction. (b) The optimized excited‐state geometries of DPh‐DPM (gas phase) and (c) its Isosurface map of interaction region indicator. (d) Frontier molecular orbitals of optimized excited‐state geometries of DPh‐DPM (gas phase), calculated by the TD‐DFT method at the B3LYP‐D3/6‐31G(d,p) level, Gaussian 09 program. DPh‐DPM, di([1,1′‐biphenyl]‐2‐yl)methane; TD‐DFT, time‐dependent density functional theory.

To prove the TSI nature of the long‐wavelength emission in DPh‐DPM, the crystal structure of DPh‐DPM was obtained and analyzed (Figure 6a). Firstly, the intermolecular interaction was investigated by mapping the Hirshfeld surface which consisted of intermolecular C⋯C, C⋯H and H⋯H interactions. Generally, only the intermolecular C⋯C interaction could affect the molecular electronic structure. However, the 2D fingerprint of the Hirshfeld surface indicated that the proportion of intermolecular C⋯C interaction was only 0.7%, suggesting the weak intermolecular TSI in DPh‐DPM. Since the intermolecular TSI was absent in DPh‐DPM crystal, it proved that the long‐wavelength CL at 390 nm came from the intramolecular TSI. Then, the single‐molecular excited‐state conformation of DPh‐DPM was optimized by the time‐dependent density functional theory (TD‐DFT) method at the B3LYP‐D3/6‐31G(d,p) level in the gas phase (Figure 6b). The results showed that the C‐C distance between two benzenes in different biphenyl moieties was very short, which was as short as 2.664 and 3.262 Å. Besides, the isosurface map of IRI for DPh‐DPM also demonstrated the strong attraction between isolated biphenyl groups (Figure 6c).^[26]^ Figure 6d showed the frontier molecular orbitals of DPh‐DPM, two isolated biphenyls had no electron overlap in HOMO, thus proving its non‐conjugation structure. However, obvious intramolecular electron overlap was observed in LUMO, demonstrating the existence of strong TSI in DPh‐DPM. Above results reveal the nature of intramolecular TSI for the CL in DPh‐DPM aggregate.

**FIGURE 7:**
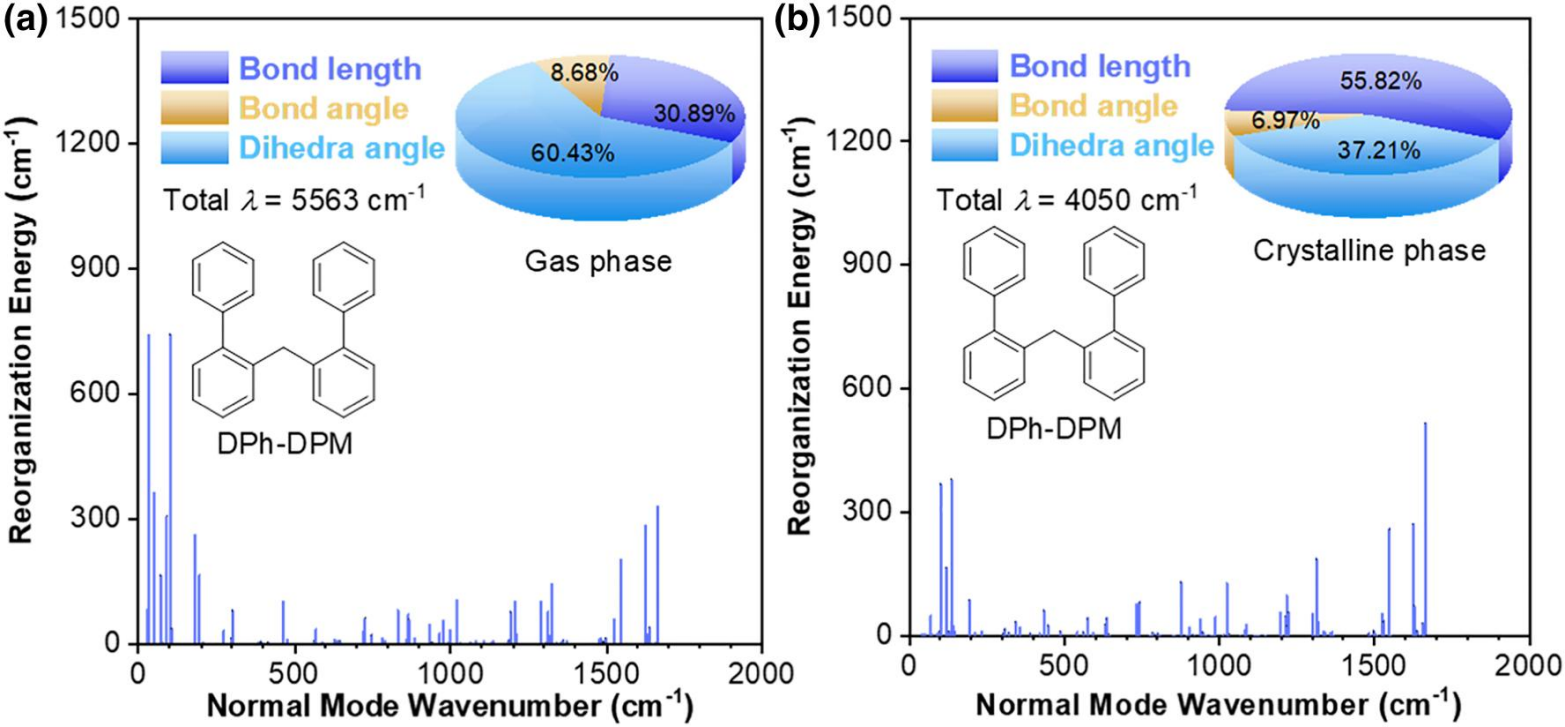
Plots of reorganization energy versus normal mode wavenumber of DPh‐DPM in (a) gas phase and (b) crystalline phase calculated by TD‐DFT, B3LYP‐D3/6‐31G(d,p), and the Gaussian 09 program. DPh‐DPM, di([1,1′‐biphenyl]‐2‐yl)methane; TD‐DFT, time‐dependent density functional theory.

Furthermore, to investigate the structural flexibility from a single molecule to aggregate, the reorganization energy in the gas phase and crystalline state was calculated by TD‐DFT, B3LYP‐D3/6‐31G(d,p), and the Gaussian 09 program (Figures 7 and S13). The results showed that the total *λ* at the single‐molecule state was 5563 cm^−1^, and 60.43% of reorganization energy came from the twisting motion of the dihedral angle located mainly in the low‐frequency region (<500 cm^−1^). However, in the crystalline state, the total λ decreased to 4050 cm^−1^ and the contribution of the low‐frequency region decreased to 37.21%, indicating that the molecular motion was greatly restricted at the aggregate state. The above calculation suggests the process of CTE in DPh‐DPM.

## 3 CONCLUSION

In this work, four DPM derivatives were designed and synthesized by changing the molecular symmetry and modifying the subunits. Experimental characterization and theoretical calculation demonstrated that DPM and DMe‐DPM almost have the same CL properties due to their similar conformation and symmetric structures. However, the asymmetric Me‐DPM which added one methyl group in the central carbon could stabilize but weaken the intramolecular TSI between two isolated phenyl rings, resulting in a stronger but blueshifted CL, respectively. Moreover, changing the bilateral phenyl rings to biphenyl rings could strengthen the intramolecular TSI and produce the redshifted CL. This work not only proves the TSI in DPM but also provides new strategies for the manipulation of TSI and CL in DPM. However, we believed that more design strategies need to be investigated to establish a more accurate relationship between the nonconjugated structure and TSI in the future.

## CONFLICT OF INTEREST STATEMENT

The authors declare that there is no conflict of interest that could be perceived as prejudicing the impartiality of the research reported.

## Supporting information

Supporting Information S1

## Data Availability

The data that supports the findings of this study are available in the supplementary material of this article.
