# Supplementary material for: Manipulation of the through‐space interactions in diphenylmethane
Source: Smart Mol. 2023 Mar 20;1(2):e20220006. doi: 10.1002/smo.20220006 (PMC12118254; doi:10.1002/smo.20220006)
Supplement: Supplementary file 1 — Supporting Information S1 [file SMO2-1-e20220006-s001.docx]

**Supporting Information**

**Manipulation of the through-space interactions in diphenylmethane**

Weihao Tu,^‡^ Zuping Xiong,^‡^ Ziteng Zhang, Jianyu Zhang, Lei Wang, Yuan Xie, Yipu Wang, Haoke Zhang,* Jing Zhi Sun,* and Ben Zhong Tang*

**Content**

[Materials 3](#_Toc120556979)

[Instruments 3](#_Toc120556980)

[Synthesis 3](#_Toc120556981)

[DPM 3](#_Toc120556982)

[DPh-DPM 3](#_Toc120556983)

[Nuclear Magnetic Resonance (NMR) Spectra 4](#_Toc120556984)

[Figure S1. ^1^H and ^13^C NMR spectra of DPM. 5](#_Toc120556985)

[Figure S2. ^1^H and ^13^C NMR spectra of DMe-DPM. 6](#_Toc120556986)

[Figure S3. ^1^H and ^13^C NMR spectra of Me-DPM. 7](#_Toc120556987)

[Figure S4. ^1^H and ^13^C NMR spectra of DPh-DPM. 8](#_Toc120556988)

[Gas chromatography Mass Spectrometry 8](#_Toc120556989)

[Figure S5. Gas chromatography mass spectrum of DPM. 8](#_Toc120556990)

[Figure S6. Gas chromatography mass spectrum of DMe-DPM. 8](#_Toc120556991)

[Figure S7. Gas chromatography mass spectrum of Me-DPM. 9](#_Toc120556992)

[Figure S8. Gas chromatography mass spectrum of DPh-DPM. 9](#_Toc120556993)

[High performance liquid chromatograph 9](#_Toc120556994)

[Figure S9. High performance liquid chromatograph of DPM. 9](#_Toc120556995)

[Figure S10. High performance liquid chromatograph of DMe-DPM. 9](#_Toc120556996)

[Figure S11. High performance liquid chromatograph of Me-DPM. 10](#_Toc120556997)

[Figure S12. High performance liquid chromatograph of DPh-DPM. 10](#_Toc120556998)

[Calculation characterizations 10](#_Toc120556999)

[Table S1. Cartesian coordinates of optimized DPM in the ground state calculated by the DFT, B3LYP-D3/6-31G(d,p), Gaussian 09 program. 10](#_Toc120557000)

[Table S2. Cartesian coordinates of optimized DPM in the excited state calculated by the DFT, B3LYP-D3/6-31G(d,p), Gaussian 09 program. 11](#_Toc120557001)

[Table S3. Cartesian coordinates of optimized DMe-DPM in the ground state calculated by the DFT, B3LYP-D3/6-31G(d,p), Gaussian 09 program. 11](#_Toc120557002)

[Table S4. Cartesian coordinates of optimized DMe-DPM in the excited state calculated by the DFT, B3LYP-D3/6-31G(d,p), Gaussian 09 program. 12](#_Toc120557003)

[Table S5. Cartesian coordinates of optimized Me-DPM in the ground state calculated by the DFT, B3LYP-D3/6-31G(d,p), Gaussian 09 program. 13](#_Toc120557004)

[Table S6. Cartesian coordinates of optimized Me-DPM in the excited state calculated by the DFT, B3LYP-D3/6-31G(d,p), Gaussian 09 program. 14](#_Toc120557005)

[Table S7. Cartesian coordinates of optimized DPh-DPM in the ground state calculated by the DFT, B3LYP-D3/6-31G(d,p), Gaussian 09 program. 14](#_Toc120557006)

[Table S8. Cartesian coordinates of optimized DPh-DPM in the excited state calculated by the DFT, B3LYP-D3/6-31G(d,p), Gaussian 09 program. 16](#_Toc120557007)

[Figure S13. Setup of QM/MM model for theoretical calculation of crystal phase, which were extracted from crystal packing structures. 17](#_Toc120557008)

Materials

Diphenylmethanol and [1,1'-biphenyl]-2-ylmethanol were purchased from macklin, ethane-1,1-diyldibenzene and propane-2,2-diyldibenzene were purchased from bidepharm. All the final products used in experiments were purified through silica gel column. Purification of all samples were checked by high performance liquid chromatography (HPLC). Acetonitrile for measurement was HPLC level from macklin.

Instruments

NMR spectra (^1^H and ^13^C) were recorded on a Bruker AVANCE NEO 600M NMR spectrometer, using deuterated dimethyl sulfoxide (DMSO-d6) as solvent. Gas Chromatography mass spectra (GC-MS) were obtained on a Agilent 8890-5977B .UV-Vis spectra were recorded on a Shimadzu UV2600 spectrometer. PL spectra were recorded on a Shimadzu RF-6000 fluorescence spectrometer. Absolute fluorescence quantum yields were measured on an Edinburgh FS5 Spectrofluorometer. Single-crystal X-ray diffraction (XRD) data were collected on a Gemini A Ultra with Atlas CCD. HPLC measurements were carried out on a WATERS 2690 liquid chromatograph with column of C18, using methanol/water mixture (ratio = 80:20, v/v).

Synthesis

DPM

1.84 g diphenylmethanol and 50 ml trifluoroacetic acid (TFA) were added into 250 ml one-necked flask, and then 3.783 g NaBH_4_ were divided into 10 parts and added to the flask^[1]^. After stirring at room temperature overnight, the reaction mixture was diluted with NaOH solution to netural pH and extracted with dichloromethane (DCM). The organic layer was separated, washed with water and brine, dried with enough anhydrous sodium sulphate. After filtration, the filtrate was evaporated under reduced pressure and the crude product was purified on a silica gel column using chloroform/hexane (1/200, v/v, Rf = 0.5). 1.31 g of DPM was obtained as colorless oliy liquid with 70.65 % yield.

DPh-DPM

2 g [1,1'-biphenyl]-2-ylmethanol **1** and 100 ml DCM were added into 250 ml two-necked flask, and the mixture was cooled into 0 ℃. Then, 4ml SOCl_2_ was added into flask. After checking the reaction with TCL, the reaction mixture was diluted with Na_2_CO_3_ solution to netural pH and extracted with dichloromethane (DCM). The organic layer was separated, washed with water and brine, dried with enough anhydrous sodium sulphate. After filtration, the filtrate was evaporated under reduced pressure and the crude product was purified on a silica gel column using chloroform/hexane (1/200, v/v, Rf = 0.6). 1.1 g of 2-(chloromethyl)-1,1'-biphenyl **2** was obtained as colorless oliy liquid with 55 % yield.

830 mg (1.1 eq) [1,1'-biphenyl]-2-ylboronic acid were added into 50 ml two-necked flask. After 3 times of nitrogen pumping and replacemen, **2** (1 eq), PdCl_2_ (1%), K_2_CO_3_ (2.6 eq), DMF (24 ml), and H_2_O (6 ml) were added and the mixture was stirred for 2h at 90 ℃. Then , 10 ml H_2_O was added to dilute the mixture and using 30 ml DCM to extract product ^[2, 3]^.The organic layer was separated, washed with water and brine, dried with enough anhydrous sodium sulphate. After filtration, the filtrate was evaporated under reduced pressure and the crude product was purified on a silica gel column using chloroform/hexane (1/200, v/v, Rf = 0.3). DPh-DPM was obtained as colorless powder with 60 % yield.

Nuclear Magnetic Resonance (NMR) Spectra


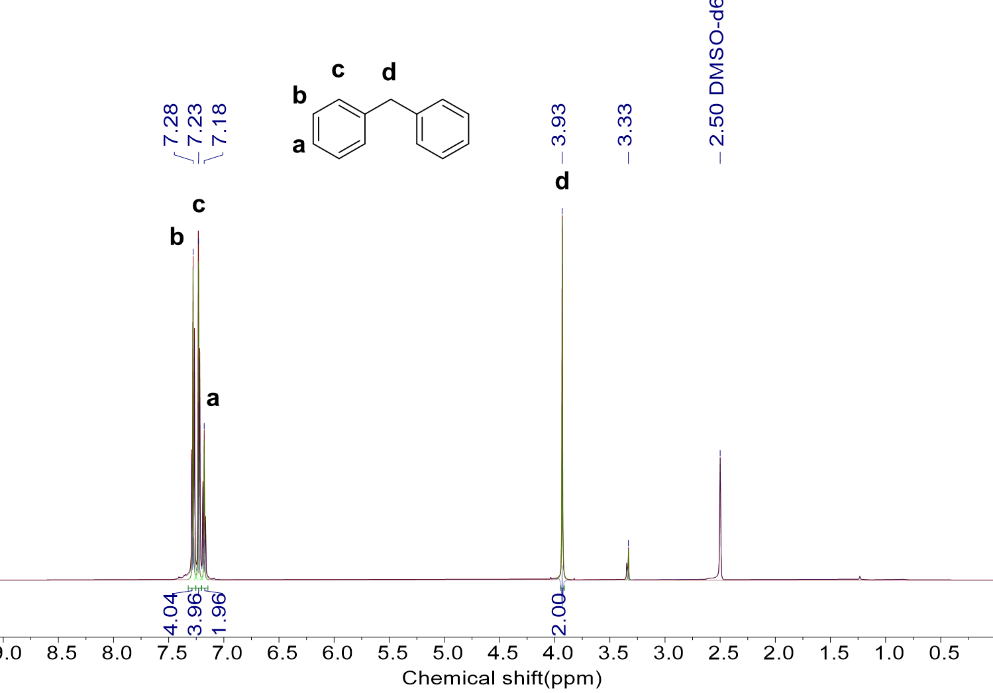


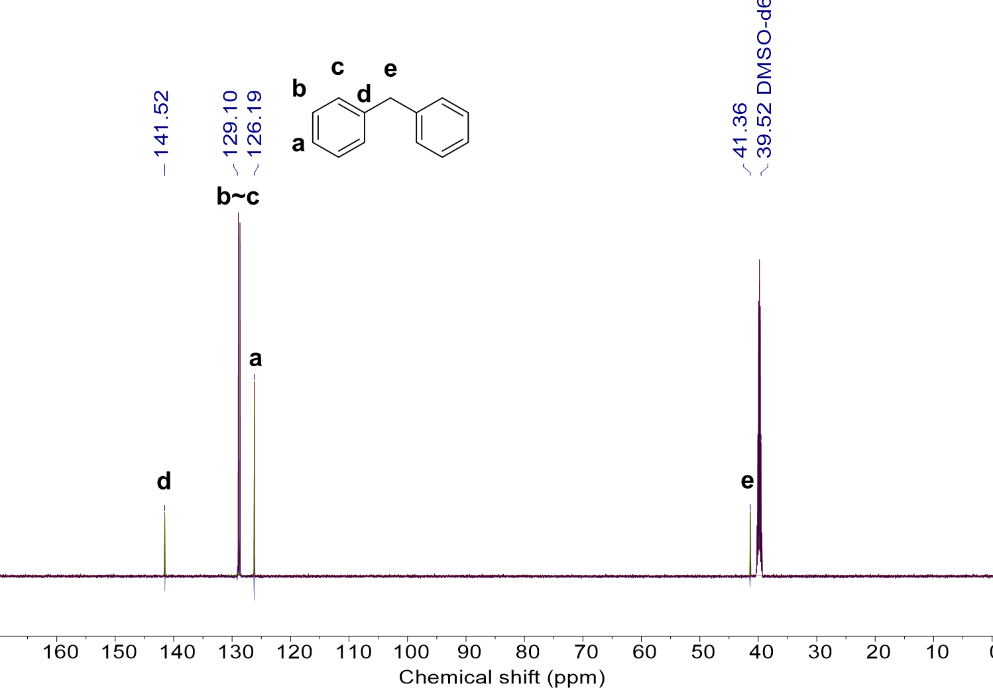


Figure S1. ^1^H and ^13^C NMR spectra of DPM.


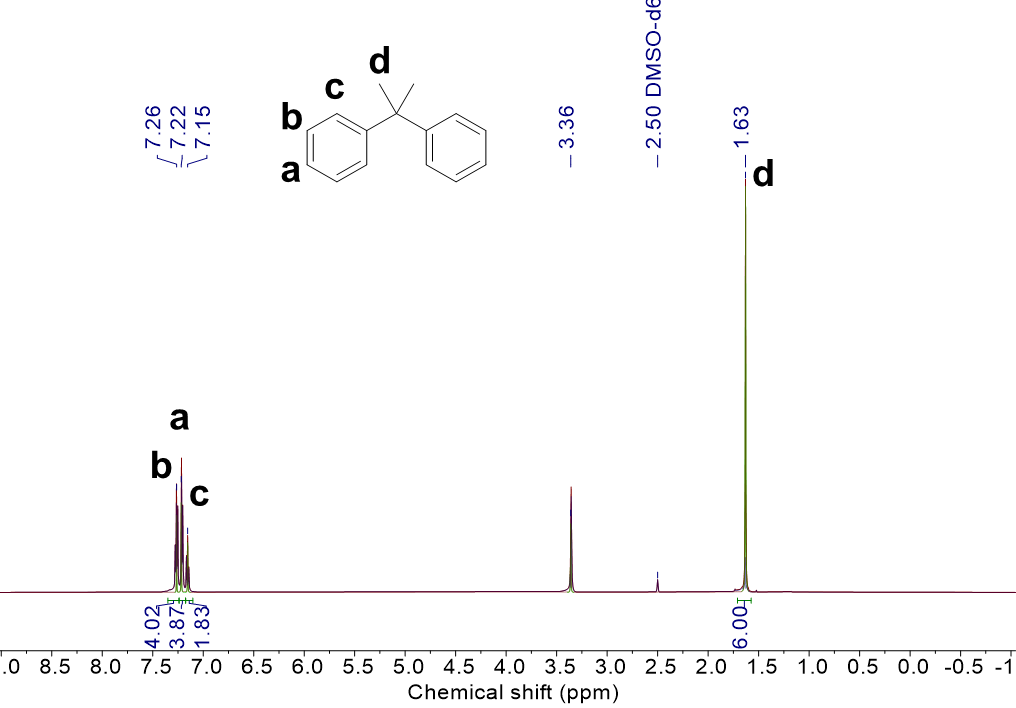


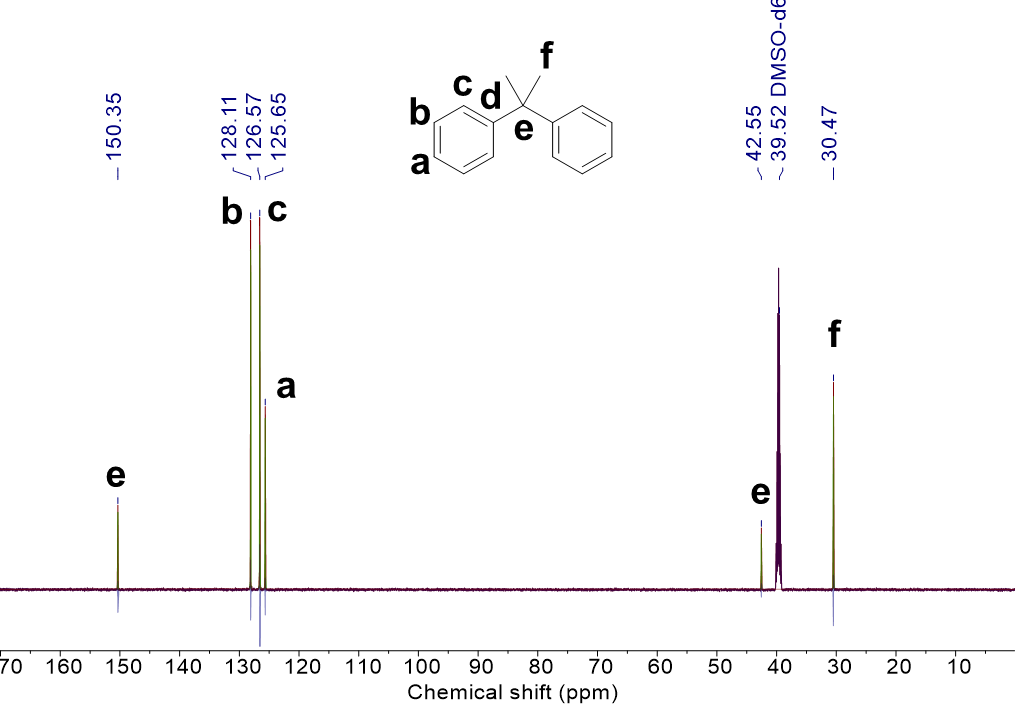


Figure S2. ^1^H and ^13^C NMR spectra of DMe-DPM.


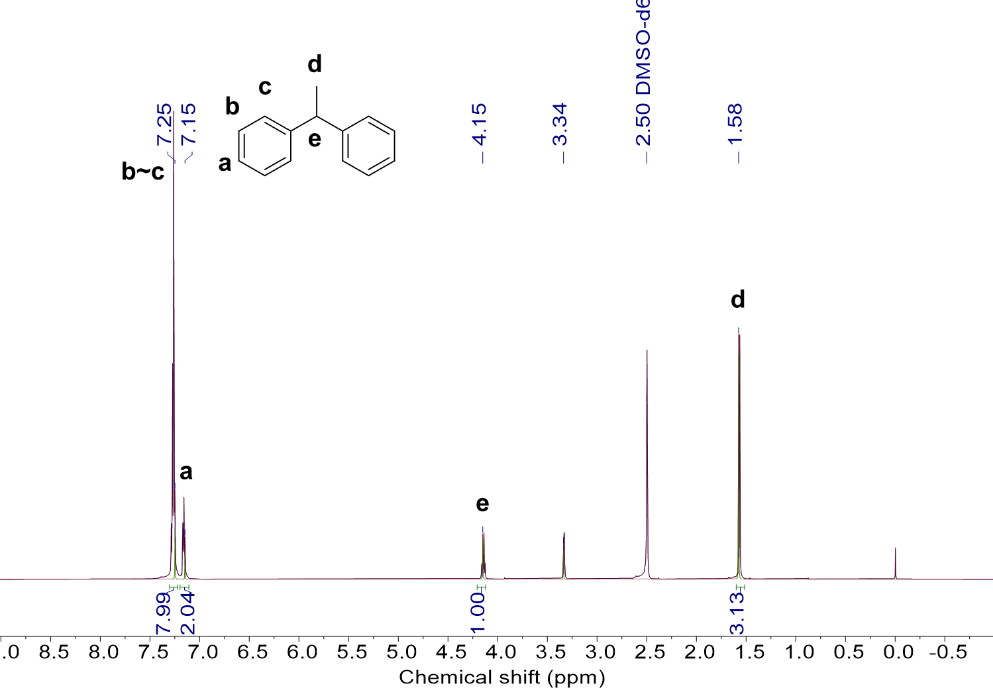


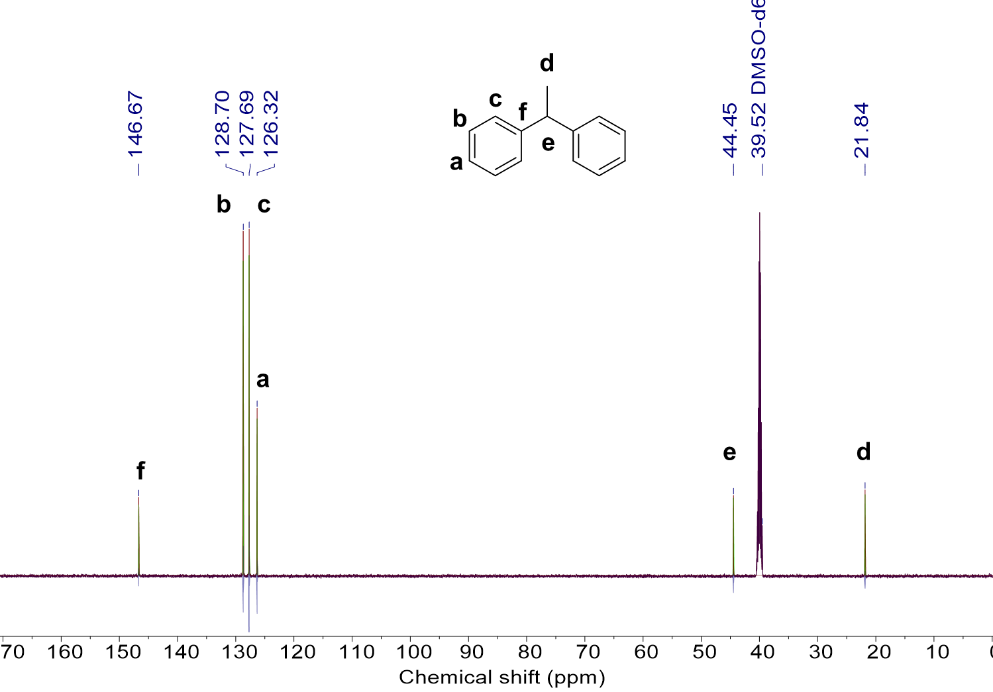


Figure S3. ^1^H and ^13^C NMR spectra of Me-DPM.


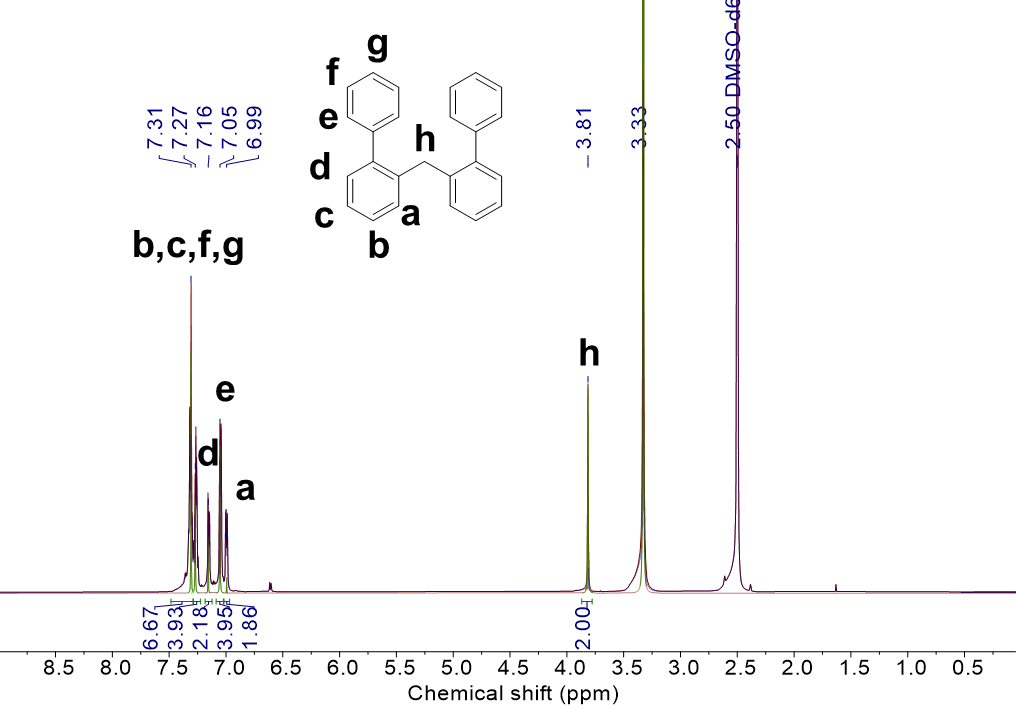


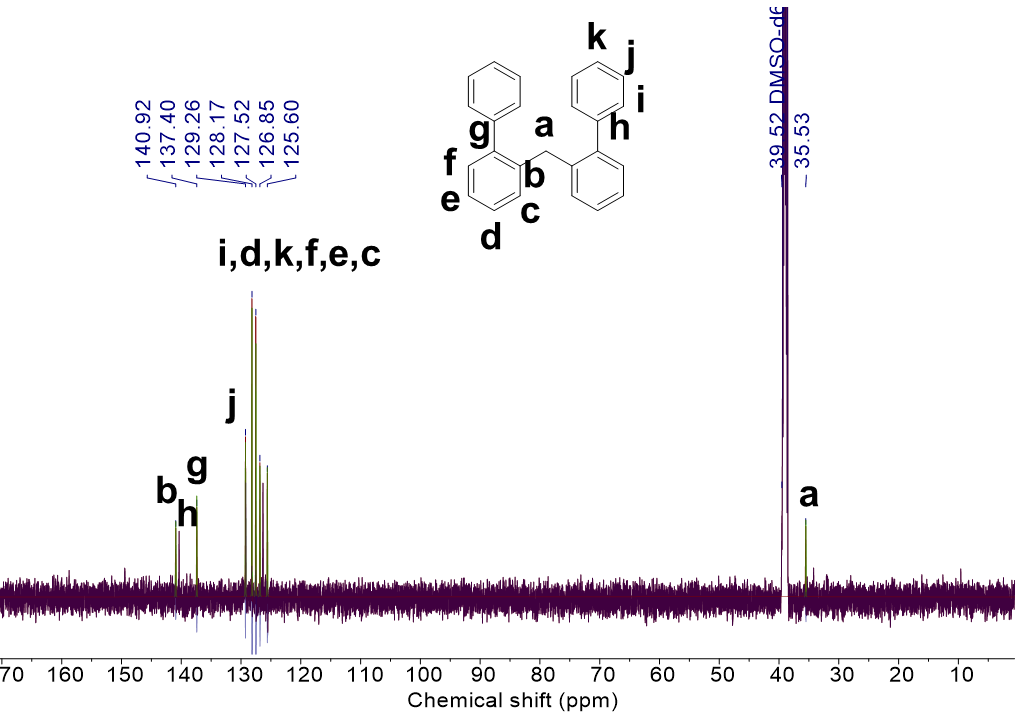


Figure S4. ^1^H and ^13^C NMR spectra of DPh-DPM.

Gas chromatography Mass Spectrometry


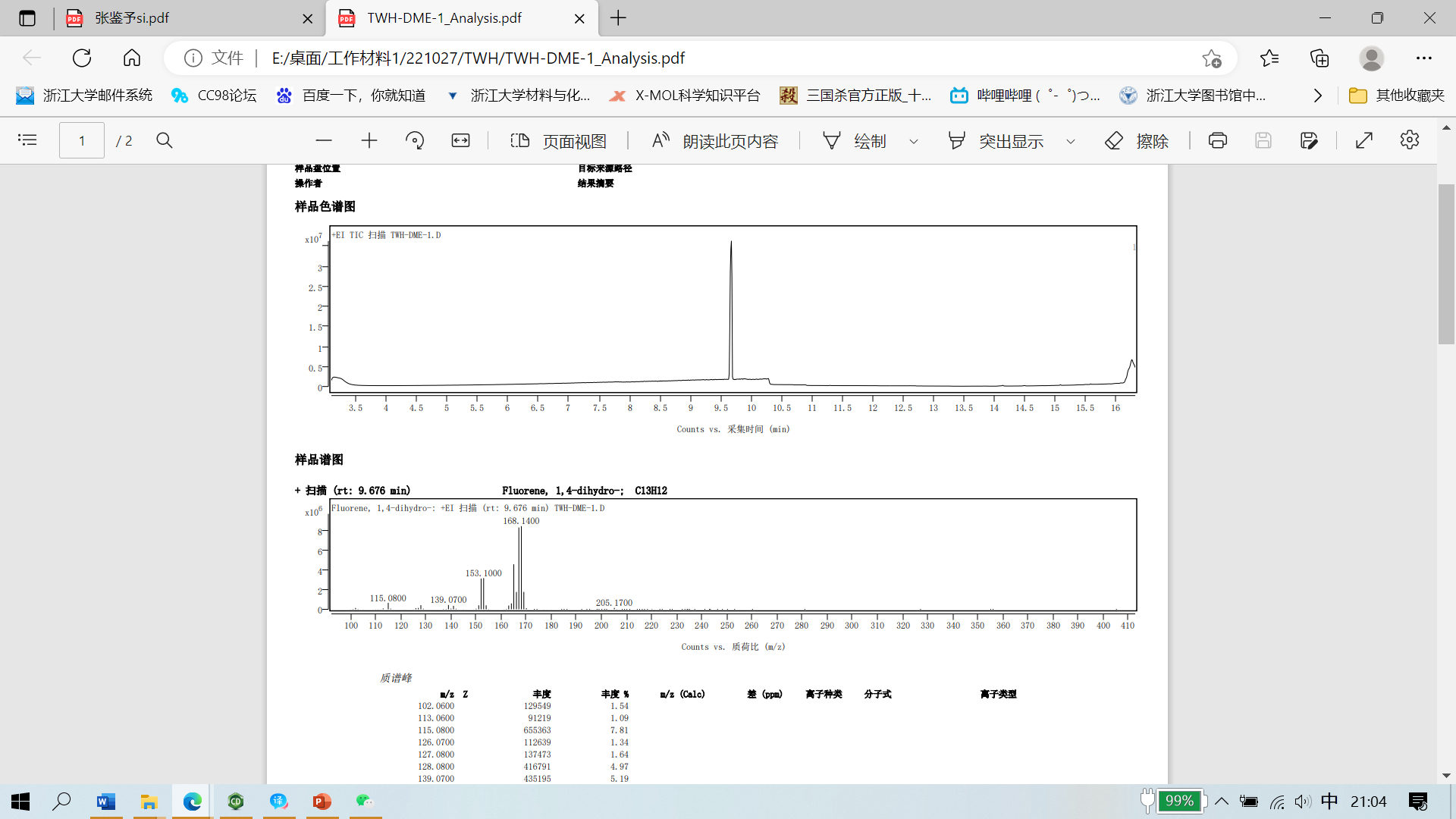


Figure S5. Gas chromatography mass spectrum of DPM.


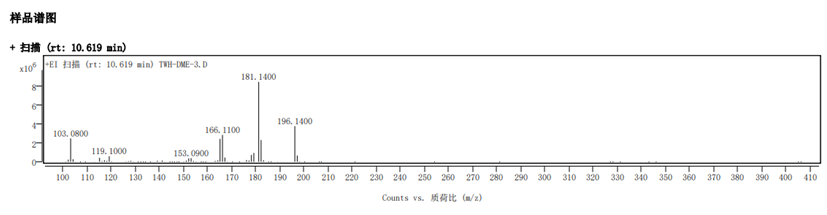


Figure S6. Gas chromatography mass spectrum of DMe-DPM.


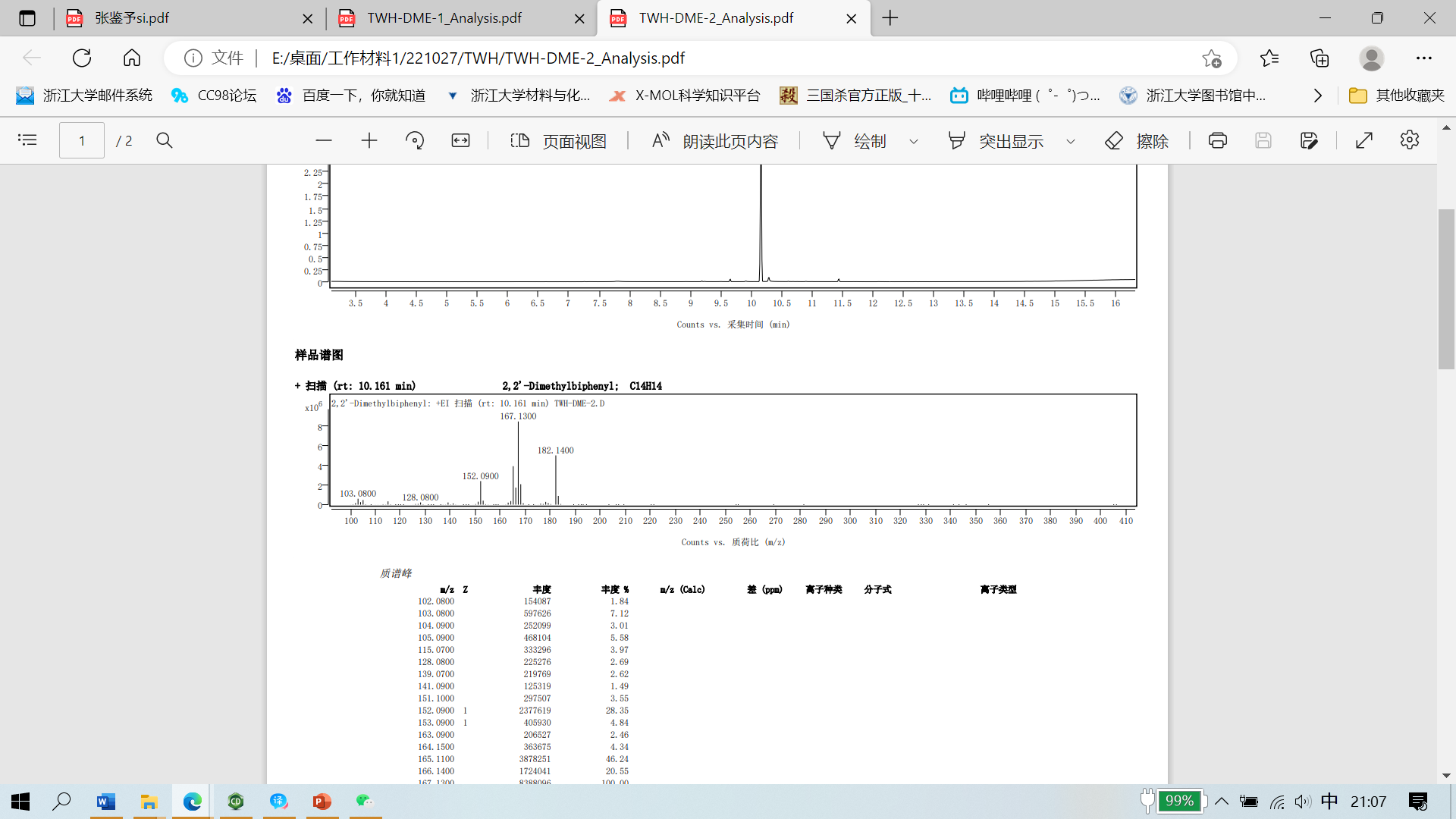


Figure S7. Gas chromatography mass spectrum of Me-DPM.


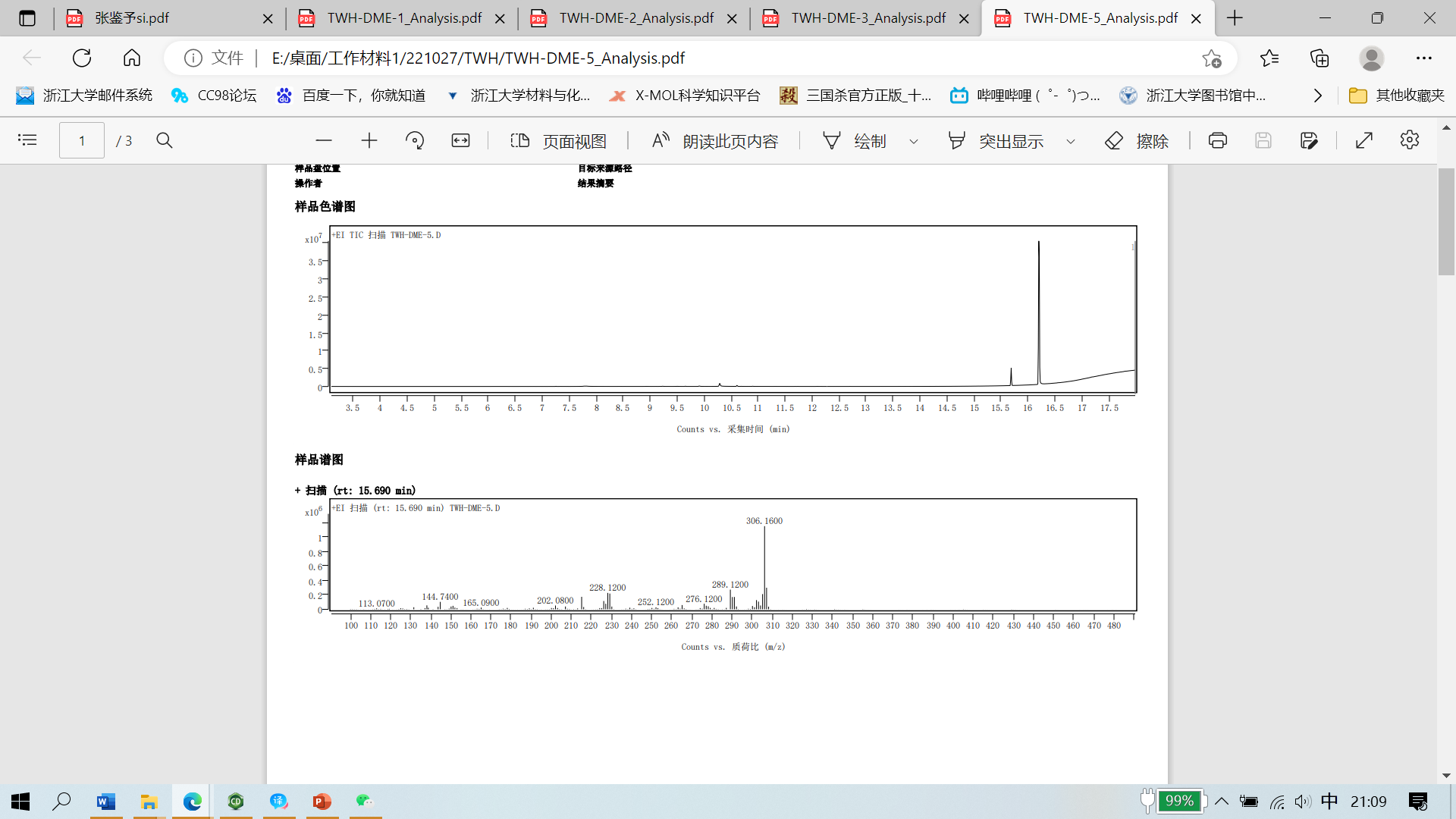


Figure S8. Gas chromatography mass spectrum of DPh-DPM.

High performance liquid chromatograph


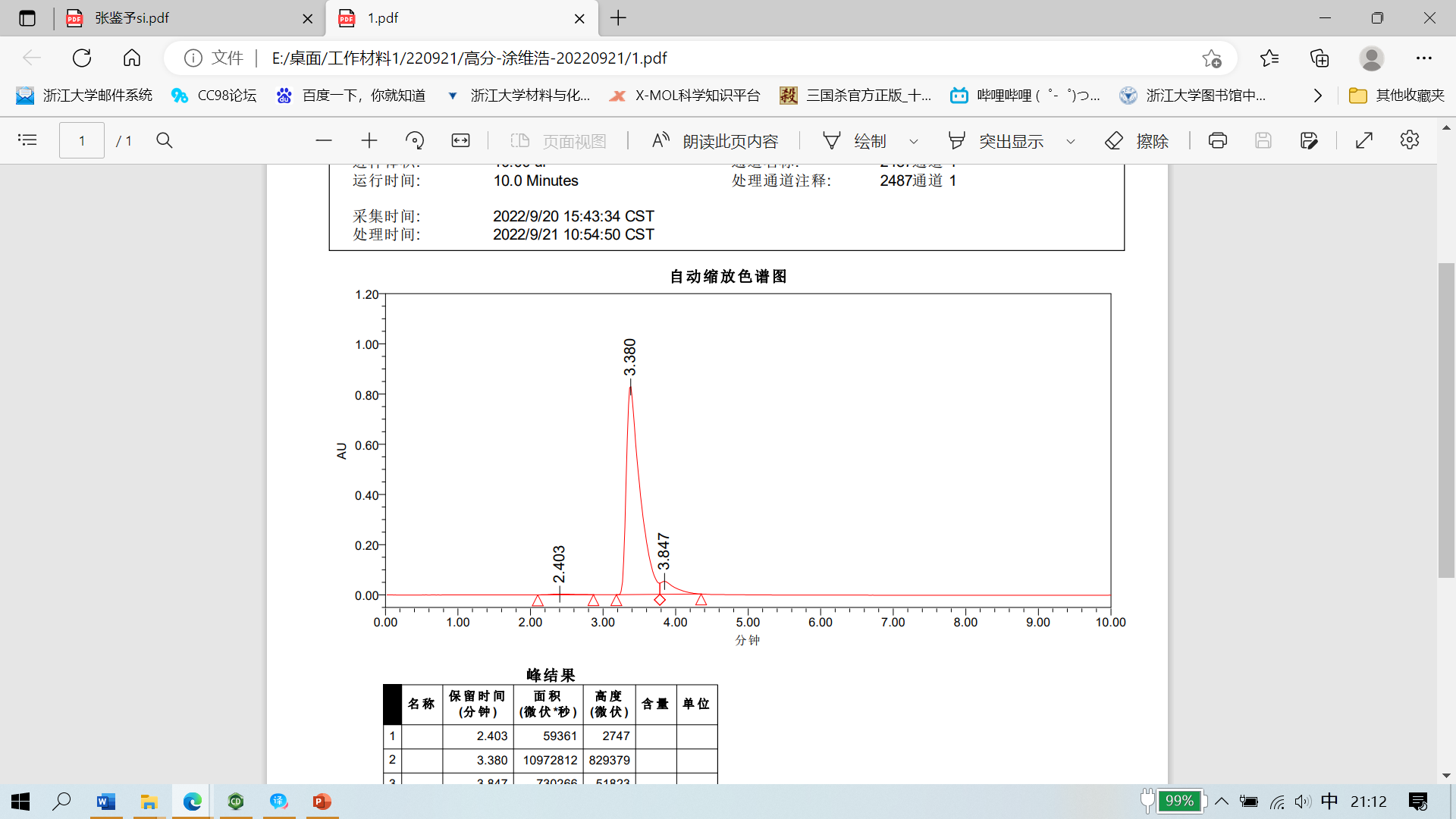


Figure S9. High performance liquid chromatograph of DPM.


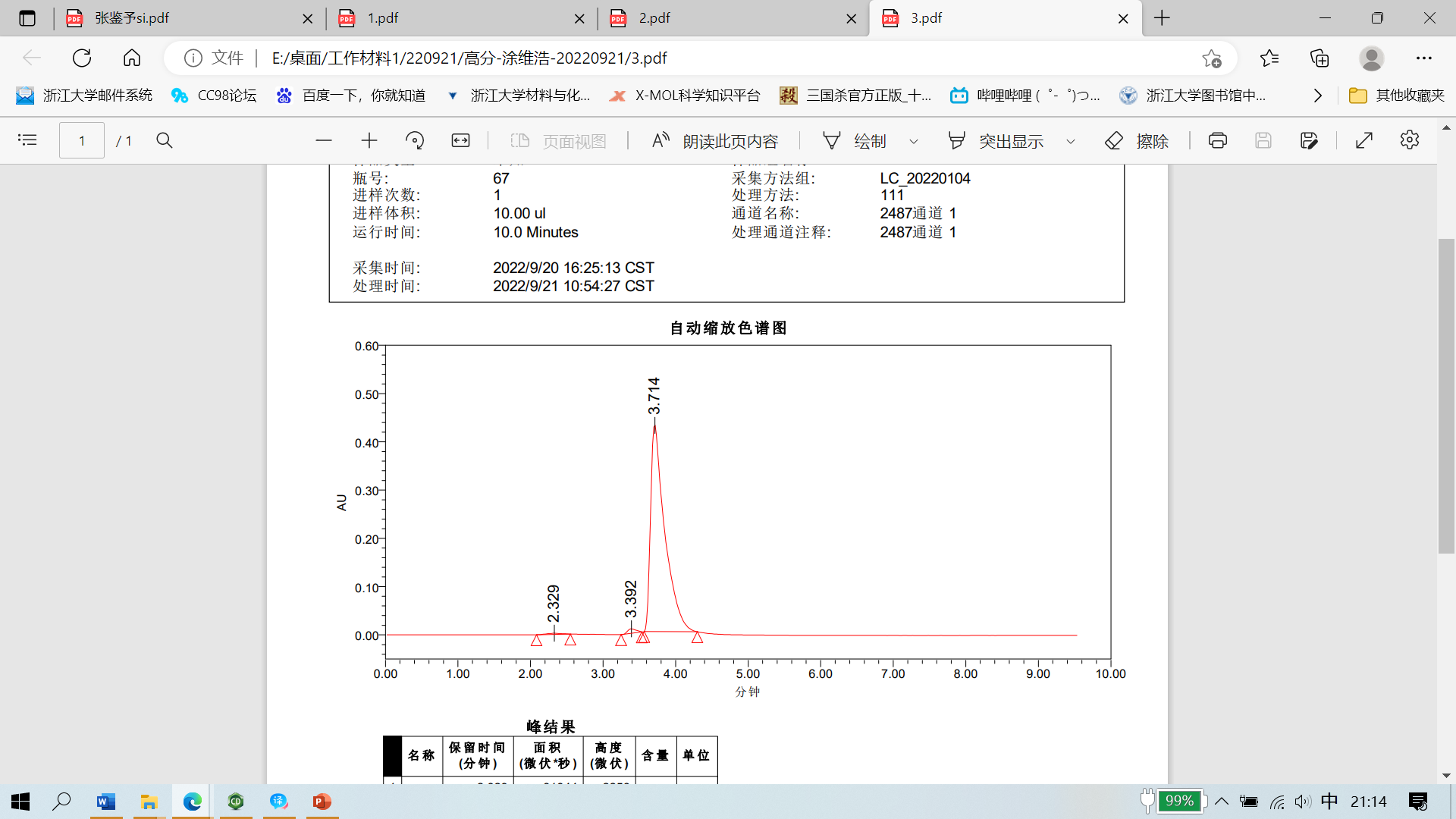


Figure S10. High performance liquid chromatograph of DMe-DPM.


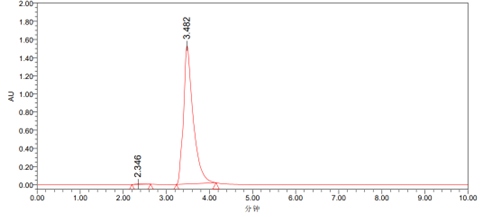


Figure S11. High performance liquid chromatograph of Me-DPM.


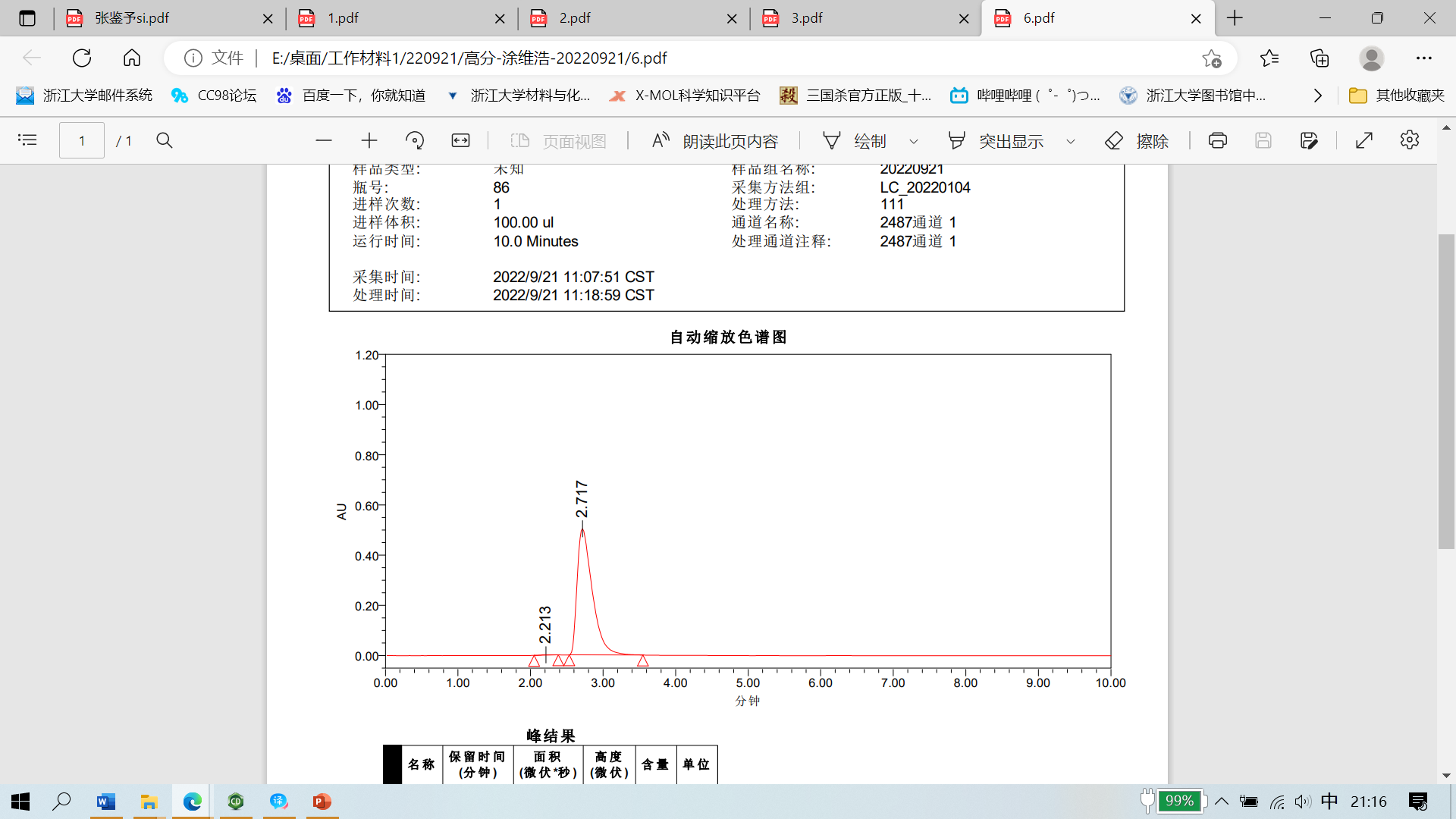


Figure S12. High performance liquid chromatograph of DPh-DPM.

Calculation characterizations

Table S1. Cartesian coordinates of optimized DPM in the ground state calculated by the DFT, B3LYP-D3/6-31G(d,p), Gaussian 09 program.

|  | X | Y | Z |
| --- | --- | --- | --- |
| C | 3.466043 | -0.07217 | -0.79664 |
| C | 3.614499 | 0.89286 | 0.197643 |
| C | 2.587702 | 1.091738 | 1.123998 |
| C | 1.424272 | 0.330081 | 1.053814 |
| C | 1.266894 | -0.64706 | 0.061881 |
| C | 2.298272 | -0.83521 | -0.86207 |
| C | -1.26689 | -0.64713 | -0.0614 |
| C | -2.29817 | -0.83445 | 0.862831 |
| C | -3.46595 | -0.07148 | 0.796826 |
| C | -3.61451 | 0.892656 | -0.19831 |
| C | -2.58781 | 1.090696 | -1.12495 |
| C | -1.42437 | 0.329103 | -1.0542 |
| C | 0.000012 | -1.47784 | 0.000555 |
| H | 4.256185 | -0.23172 | -1.52437 |
| H | 4.52055 | 1.488721 | 0.250509 |
| H | 2.693931 | 1.84428 | 1.899777 |
| H | 0.620713 | 0.497099 | 1.765526 |
| H | 2.186367 | -1.58647 | -1.63954 |
| H | -2.18618 | -1.58502 | 1.640963 |
| H | -4.25602 | -0.23037 | 1.524785 |
| H | -4.52057 | 1.488469 | -0.25162 |
| H | -2.69412 | 1.842534 | -1.9014 |
| H | -0.62089 | 0.495475 | -1.76615 |
| H | 0.050231 | -2.13724 | -0.87434 |
| H | -0.05019 | -2.13658 | 0.875953 |

Table S2. Cartesian coordinates of optimized DPM in the excited state calculated by the DFT, B3LYP-D3/6-31G(d,p), Gaussian 09 program.

|  | X | Y | Z |
| --- | --- | --- | --- |
| C | 2.542315 | -1.22471 | -0.67924 |
| C | 2.998331 | 0.000489 | -1.19628 |
| C | 2.542049 | 1.225278 | -0.6785 |
| C | 1.591333 | 1.228325 | 0.360941 |
| C | 1.134897 | -0.00035 | 0.901143 |
| C | 1.59159 | -1.22859 | 0.360196 |
| C | -1.1349 | -0.00035 | 0.901143 |
| C | -1.59139 | 1.22831 | 0.360975 |
| C | -2.54211 | 1.225239 | -0.67846 |
| C | -2.99833 | 0.000443 | -1.19628 |
| C | -2.54225 | -1.22475 | -0.67927 |
| C | -1.59153 | -1.2286 | 0.360162 |
| C | 0.000000 | -0.00075 | 1.891318 |
| H | 2.915397 | -2.15948 | -1.08237 |
| H | 3.719389 | 0.000811 | -2.00855 |
| H | 2.914927 | 2.160366 | -1.08107 |
| H | 1.235785 | 2.160671 | 0.783746 |
| H | 1.236249 | -2.16128 | 0.782421 |
| H | -1.23591 | 2.160671 | 0.783801 |
| H | -2.91504 | 2.16032 | -1.08101 |
| H | -3.71939 | 0.000754 | -2.00855 |
| H | -2.91528 | -2.15952 | -1.08243 |
| H | -1.23612 | -2.16128 | 0.782366 |
| H | 0.000003 | -0.89304 | 2.521934 |
| H | -3E-06 | 0.891067 | 2.522614 |

Table S3. Cartesian coordinates of optimized DMe-DPM in the ground state calculated by the DFT, B3LYP-D3/6-31G(d,p), Gaussian 09 program.

|  | X | Y | Z |
| --- | --- | --- | --- |
| C | -2.4656 | -1.53434 | 1.09159 |
| C | -3.54618 | -1.33707 | 0.228651 |
| C | -3.4772 | -0.32966 | -0.72979 |
| C | -2.34049 | 0.477944 | -0.82541 |
| C | -1.25244 | 0.292115 | 0.032392 |
| C | -1.3348 | -0.73012 | 0.990493 |
| C | 1.252438 | 0.292111 | -0.03239 |
| C | 1.334791 | -0.73013 | -0.99048 |
| C | 2.465594 | -1.53435 | -1.09158 |
| C | 3.546184 | -1.33707 | -0.22865 |
| C | 3.477206 | -0.32965 | 0.729783 |
| C | 2.340493 | 0.477948 | 0.825404 |
| C | 8.7E-07 | 1.181354 | -1.1E-06 |
| C | 0.046175 | 2.091405 | -1.24626 |
| C | -0.04617 | 2.091408 | 1.24625 |
| H | -2.50245 | -2.32003 | 1.840437 |
| H | -4.42848 | -1.96545 | 0.302125 |
| H | -4.30756 | -0.16736 | -1.41077 |
| H | -2.3128 | 1.253997 | -1.58131 |
| H | -0.49131 | -0.90378 | 1.651795 |
| H | 0.491302 | -0.9038 | -1.65178 |
| H | 2.502441 | -2.32005 | -1.84042 |
| H | 4.428486 | -1.96545 | -0.30213 |
| H | 4.307571 | -0.16735 | 1.41076 |
| H | 2.312801 | 1.254004 | 1.5813 |
| H | 0.98039 | 2.659275 | -1.24614 |
| H | 0.007761 | 1.511062 | -2.17179 |
| H | -0.78226 | 2.806624 | -1.24936 |
| H | 0.782278 | 2.806608 | 1.249361 |
| H | -0.00778 | 1.511066 | 2.171788 |
| H | -0.98037 | 2.659299 | 1.246115 |

Table S4. Cartesian coordinates of optimized DMe-DPM in the excited state calculated by the DFT, B3LYP-D3/6-31G(d,p), Gaussian 09 program.

|  | X | Y | Z |
| --- | --- | --- | --- |
| C | -2.2439 | -1.62821 | 1.084175 |
| C | -3.37503 | -1.43775 | 0.273496 |
| C | -3.42692 | -0.38 | -0.65178 |
| C | -2.33648 | 0.507599 | -0.77781 |
| C | -1.20749 | 0.353718 | 0.052946 |
| C | -1.15844 | -0.73405 | 0.969732 |
| C | 1.207516 | 0.353705 | -0.05297 |
| C | 1.158328 | -0.73421 | -0.96957 |
| C | 2.24376 | -1.62842 | -1.084 |
| C | 3.374983 | -1.43785 | -0.27348 |
| C | 3.427004 | -0.37997 | 0.65163 |
| C | 2.336602 | 0.50768 | 0.777646 |
| C | 0.00002 | 1.277592 | -6E-06 |
| C | 0.010026 | 2.190295 | -1.24941 |
| C | -0.00998 | 2.190293 | 1.249398 |
| H | -2.20397 | -2.44921 | 1.790708 |
| H | -4.22273 | -2.11079 | 0.362927 |
| H | -4.30689 | -0.24656 | -1.27214 |
| H | -2.37366 | 1.302771 | -1.51089 |
| H | -0.30042 | -0.82634 | 1.626603 |
| H | 0.300226 | -0.82659 | -1.62632 |
| H | 2.203725 | -2.44953 | -1.7904 |
| H | 4.222653 | -2.11094 | -0.3629 |
| H | 4.307044 | -0.24646 | 1.271869 |
| H | 2.373872 | 1.302961 | 1.510598 |
| H | 0.935434 | 2.771258 | -1.26483 |
| H | -0.04353 | 1.608408 | -2.17206 |
| H | -0.83146 | 2.88939 | -1.22531 |
| H | 0.831575 | 2.889308 | 1.225361 |
| H | 0.043454 | 1.608395 | 2.172051 |
| H | -0.93534 | 2.771342 | 1.264759 |

Table S5. Cartesian coordinates of optimized Me-DPM in the ground state calculated by the DFT, B3LYP-D3/6-31G(d,p), Gaussian 09 program.

|  | X | Y | Z |
| --- | --- | --- | --- |
| C | -2.6577 | -1.48868 | -0.89393 |
| C | -3.59129 | -1.12469 | 0.080109 |
| C | -3.35961 | 0.004504 | 0.861027 |
| C | -2.20454 | 0.768634 | 0.671488 |
| C | -1.26368 | 0.414718 | -0.29931 |
| C | -1.50807 | -0.72703 | -1.07663 |
| C | 1.25064 | 0.362018 | -0.18965 |
| C | 2.352894 | 0.329014 | -1.04901 |
| C | 3.499258 | -0.39388 | -0.71574 |
| C | 3.556642 | -1.09538 | 0.487824 |
| C | 2.460407 | -1.07034 | 1.352469 |
| C | 1.317114 | -0.34936 | 1.014687 |
| C | 0.023084 | 1.195946 | -0.53726 |
| C | 0.084595 | 2.549262 | 0.188465 |
| H | -2.827 | -2.36735 | -1.50931 |
| H | -4.48924 | -1.71725 | 0.226055 |
| H | -4.07756 | 0.297717 | 1.621357 |
| H | -2.04574 | 1.64654 | 1.287678 |
| H | -0.77728 | -1.02068 | -1.82551 |
| H | 2.311669 | 0.874841 | -1.98837 |
| H | 4.34401 | -0.41137 | -1.39806 |
| H | 4.445612 | -1.66144 | 0.748819 |
| H | 2.494438 | -1.61883 | 2.28922 |
| H | 0.459482 | -0.34706 | 1.680834 |
| H | 0.073418 | 1.403109 | -1.61419 |
| H | 1.001061 | 3.076749 | -0.0884 |
| H | -0.77055 | 3.180725 | -0.0719 |
| H | 0.099205 | 2.41582 | 1.274492 |

Table S6. Cartesian coordinates of optimized Me-DPM in the excited state calculated by the DFT, B3LYP-D3/6-31G(d,p), Gaussian 09 program.

|  | X | Y | Z |
| --- | --- | --- | --- |
| C | -1.85516 | -1.78869 | -0.57014 |
| C | -3.08043 | -1.46972 | -0.02569 |
| C | -3.38884 | -0.12539 | 0.333252 |
| C | -2.435 | 0.879297 | 0.174582 |
| C | -1.18603 | 0.602205 | -0.37429 |
| C | -0.8707 | -0.77913 | -0.76521 |
| C | 1.184988 | 0.643639 | -0.01644 |
| C | 2.452218 | 0.811613 | -0.56244 |
| C | 3.445274 | -0.1518 | -0.38032 |
| C | 3.137314 | -1.362 | 0.305409 |
| C | 1.885694 | -1.57505 | 0.842037 |
| C | 0.869722 | -0.58244 | 0.71299 |
| C | 0.000387 | 1.514237 | -0.36827 |
| C | -0.13441 | 2.768811 | 0.512569 |
| H | -1.63599 | -2.80817 | -0.87295 |
| H | -3.82975 | -2.24295 | 0.116278 |
| H | -4.36338 | 0.111121 | 0.747506 |
| H | -2.66216 | 1.885989 | 0.511836 |
| H | -0.10985 | -0.92669 | -1.53365 |
| H | 2.657465 | 1.699026 | -1.15885 |
| H | 4.444471 | 0.009094 | -0.77121 |
| H | 3.909423 | -2.11818 | 0.41726 |
| H | 1.668899 | -2.49101 | 1.383623 |
| H | 0.075626 | -0.5343 | 1.461454 |
| H | 0.190037 | 1.856407 | -1.40334 |
| H | 0.767509 | 3.380644 | 0.437532 |
| H | -0.98704 | 3.377878 | 0.196736 |
| H | -0.27542 | 2.487562 | 1.559591 |

Table S7. Cartesian coordinates of optimized DPh-DPM in the ground state calculated by the DFT, B3LYP-D3/6-31G(d,p), Gaussian 09 program.

|  | X | Y | Z |
| --- | --- | --- | --- |
| C | 2.027855 | -2.13447 | 1.388589 |
| C | 1.239611 | -3.01874 | 2.120809 |
| C | -0.10346 | -3.177 | 1.783806 |
| C | -0.63936 | -2.45206 | 0.721227 |
| C | 0.138444 | -1.5528 | -0.01717 |
| C | 1.497172 | -1.39022 | 0.325264 |
| C | -1.94929 | -0.53006 | -1.07899 |
| C | -2.88231 | -1.30885 | -1.77159 |
| C | -4.25087 | -1.05593 | -1.68315 |
| C | -4.70722 | -0.00136 | -0.89545 |
| C | -3.78908 | 0.785122 | -0.20066 |
| C | -2.41486 | 0.530094 | -0.27478 |
| C | -0.47185 | -0.82462 | -1.20758 |
| C | 2.405734 | -0.46122 | -0.40544 |
| C | -1.47545 | 1.406115 | 0.486751 |
| C | 3.514633 | -0.96397 | -1.09915 |
| C | 4.391586 | -0.10361 | -1.76021 |
| C | 4.173383 | 1.273955 | -1.7344 |
| C | 3.076078 | 1.784924 | -1.03904 |
| C | 2.200734 | 0.926075 | -0.37806 |
| C | -1.15482 | 2.681538 | 0.00327 |
| C | -0.29576 | 3.518711 | 0.716452 |
| C | 0.250159 | 3.091738 | 1.927648 |
| C | -0.06601 | 1.824086 | 2.419313 |
| C | -0.92099 | 0.986379 | 1.703999 |
| H | 3.0731 | -1.99446 | 1.647297 |
| H | 1.670422 | -3.57551 | 2.947371 |
| H | -0.73332 | -3.86242 | 2.343011 |
| H | -1.685 | -2.57697 | 0.459311 |
| H | -2.52382 | -2.12352 | -2.39519 |
| H | -4.95362 | -1.67589 | -2.23149 |
| H | -5.76944 | 0.210512 | -0.82153 |
| H | -4.13387 | 1.609797 | 0.415912 |
| H | 0.070306 | 0.105463 | -1.38122 |
| H | -0.31128 | -1.43676 | -2.10434 |
| H | 3.680359 | -2.03695 | -1.12331 |
| H | 5.243447 | -0.5104 | -2.29714 |
| H | 4.854888 | 1.94415 | -2.24961 |
| H | 2.901023 | 2.85607 | -1.00214 |
| H | 1.363105 | 1.329771 | 0.175285 |
| H | -1.5804 | 3.010736 | -0.9398 |
| H | -0.05383 | 4.502637 | 0.325953 |
| H | 0.921095 | 3.7402 | 2.482495 |
| H | 0.358866 | 1.482249 | 3.357942 |
| H | -1.15629 | -0.00168 | 2.082361 |

Table S8. Cartesian coordinates of optimized DPh-DPM in the excited state calculated by the DFT, B3LYP-D3/6-31G(d,p), Gaussian 09 program.

|  | X | Y | Z |
| --- | --- | --- | --- |
| C | 1.927329 | -2.43237 | 0.567356 |
| C | 1.168336 | -3.45532 | 1.085969 |
| C | -0.24085 | -3.49489 | 0.884642 |
| C | -0.82376 | -2.48302 | 0.147978 |
| C | -0.09087 | -1.39619 | -0.37809 |
| C | 1.367019 | -1.32977 | -0.1509 |
| C | -2.19899 | -0.14075 | -1.0226 |
| C | -3.29876 | -0.48509 | -1.81414 |
| C | -4.58095 | -0.02299 | -1.51767 |
| C | -4.78152 | 0.813338 | -0.41925 |
| C | -3.69894 | 1.17002 | 0.381899 |
| C | -2.41358 | 0.680921 | 0.11344 |
| C | -0.80339 | -0.57037 | -1.42332 |
| C | 2.229745 | -0.27379 | -0.6057 |
| C | -1.27557 | 1.075515 | 0.973499 |
| C | 3.618397 | -0.52734 | -0.86864 |
| C | 4.467423 | 0.464311 | -1.31037 |
| C | 4.007734 | 1.785209 | -1.51567 |
| C | 2.665514 | 2.069396 | -1.25867 |
| C | 1.796442 | 1.083782 | -0.80996 |
| C | -0.80366 | 2.391024 | 0.955137 |
| C | 0.398455 | 2.709026 | 1.601837 |
| C | 1.107665 | 1.724927 | 2.313386 |
| C | 0.602531 | 0.437898 | 2.418011 |
| C | -0.5986 | 0.10791 | 1.760857 |
| H | 2.989954 | -2.41657 | 0.778997 |
| H | 1.651739 | -4.23847 | 1.662918 |
| H | -0.83409 | -4.31531 | 1.272518 |
| H | -1.88965 | -2.51527 | -0.06132 |
| H | -3.14026 | -1.11531 | -2.68446 |
| H | -5.41806 | -0.30879 | -2.14702 |
| H | -5.77558 | 1.179674 | -0.1829 |
| H | -3.84332 | 1.812385 | 1.244914 |
| H | -0.2136 | 0.311031 | -1.69114 |
| H | -0.86832 | -1.14982 | -2.35553 |
| H | 3.99729 | -1.5395 | -0.78147 |
| H | 5.504204 | 0.218301 | -1.52381 |
| H | 4.686799 | 2.558853 | -1.8579 |
| H | 2.288752 | 3.081559 | -1.38278 |
| H | 0.793842 | 1.36771 | -0.54677 |
| H | -1.32007 | 3.134052 | 0.356778 |
| H | 0.80152 | 3.713604 | 1.527298 |
| H | 2.048179 | 1.983407 | 2.788588 |
| H | 1.122223 | -0.32055 | 2.990924 |
| H | -1.06449 | -0.84934 | 1.938038 |


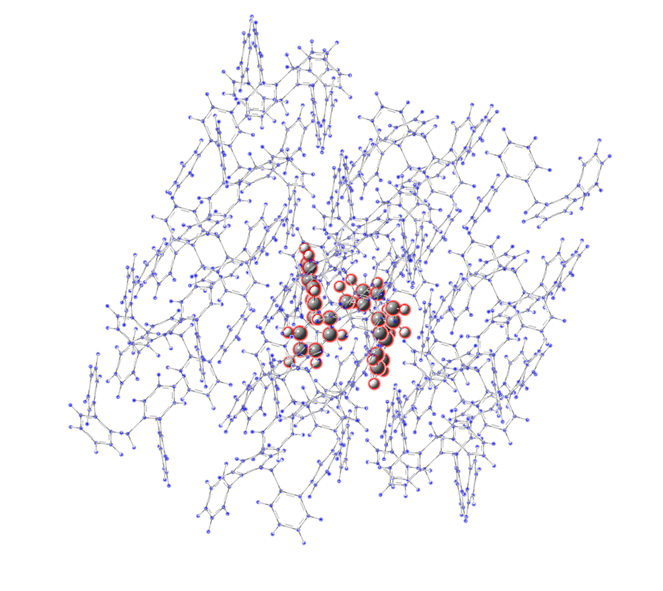


Figure S13. Setup of QM/MM model for theoretical calculation of crystal phase, which were extracted from crystal packing structures.

**References:**

[1]. Herrero García, N., I. Fernández and J. Osío Barcina, Electron Delocalization in Homoconjugated 7,7‐Diarylnorbornane Systems: A Computational and Experimental Study. Chemistry – A European Journal, 2011. 17(26): p. 7327-7335.

[2]. Zhao, G., et al., Efficient synthesis of diarylmethane derivatives by PdCl 2 catalyzed cross-coupling reactions of benzyl chlorides with aryl boronic acids in aqueous medium. Tetrahedron Letters, 2015. 56(48): p. 6700-6703.

[3]. Bandgar, B.P., S.V. Bettigeri and J. Phopase, Palladium catalyzed ligand-free Suzuki cross-coupling reactions of benzylic halides with aryl boronic acids under mild conditions. Tetrahedron Letters, 2004. 45(37): p. 6959-6962.
